# Multiple micronutrient supplementation using spirulina platensis and infant growth, morbidity, and motor development: Evidence from a randomized trial in Zambia

**DOI:** 10.1371/journal.pone.0211693

**Published:** 2019-02-13

**Authors:** Kazuya Masuda, Maureen Chitundu

**Affiliations:** 1 Institute of Economic Research, Hitotsubashi University, Tokyo, Japan; 2 Programme Against Malnutrition, Lusaka, Zambia; TNO, NETHERLANDS

## Abstract

In developing countries, micronutrient deficiency in infants is associated with growth faltering, morbidity, and delayed motor development. One of the potentially low-cost and sustainable solutions is to use locally producible food for the home fortification of complementary foods. This study aimed to test the hypothesis that locally producible spirulina platensis supplementation would achieve the following: 1) increase infant physical growth, 2) reduce morbidity, and 3) improve motor development. We randomly assigned 501 Zambian infants into the control group or the spirulina group. Children in the control group (n = 250) received a soya-maize-based porridge for 12 months; those in the spirulina group (n = 251) received the same food with the addition of spirulina. We assessed the change in infants’ anthropometric status, morbidity (probable pneumonia, cough, probable malaria, and fever), and motor development over 12 months. The baseline characteristics were not different between the two groups. The attrition rate (47/501) was low. The physical growth of infants in the two groups was similar at 12 months of intervention, as measured by height-for-age z-scores and weight-for-age z-scores. Infants in the spirulina group were 11 percentage points less likely to develop a cough (CI: -0.23, -0.00; P < 0.05) and were more likely to be able to walk alone at 15 months (0.96 ± 0.19) than infants in the control group (0.92 ± 0.28). Home-fortification of complementary foods using spirulina had positive effects on upper respiratory infection morbidity prevention and motor milestone acquisition among Zambian infants.

## Introduction

Micronutrient deficiency in infancy is associated with growth faltering [1], morbidity [2], and delayed motor development [3], and is common in developing countries where the food available for infants has low micronutrient density [4]. A low-cost and sustainable way to address this problem is to utilize locally producible foods rich in multi-micronutrients as home supplements to complementary food. *Arthrospira platensis*, also known as spirulina, is a blue-green micro-algae, from the Oscillatoriaceae family, indigenous to Africa [5, 6]. It contains a high percentage of protein, and is rich in multiple micronutrients (MMN) known to support infant growth, such as beta carotene, B vitamins, and minerals including calcium, iron, magnesium, manganese, potassium, and zinc [6–9]. Other than breast milk, spirulina is the only dietary source of gamma linolenic acid, and contains various other essential fatty acids and amino acids [10, 11]. The cost of producing spirulina is much lower than that of producing other comparably protein-rich foods, such as soya beans or beef [12], and therefore may potentially be a sustainable method for meeting the nutritional demands of African infants.

Spirulina has been shown to be effective in treating female anemia [13–15], malnutrition in adults [16], and growth faltering in children who are malnourished [17–20] or infected with HIV [21]. To date, however, no study has explored the association between daily spirulina supplementation and growth and development in children aged below two years. Our objective was to assess the acceptability and effects of spirulina supplementation on growth, incidence of morbidity, and level of motor development in infants in Zambia. The testable hypothesis was that spirulina supplementation for 12 months would increase infant height, reduce the incidence of morbidity, and reduce time taken to achieve motor development milestones. Zambia provides an appropriate setting to test this hypothesis because micronutrient deficiency and chronic malnutrition (stunting) is highly prevalent in the country [22, 23], and spirulina can be produced locally. Indeed, on average, 20% of children under five are stunted, while 15% are underweight in Zambia [22]. Furthermore, geographic variation in its prevalence exists, and the statistics for our study area, Luapula province, are markedly above average; 43% of children are stunted, 22.2% are underweight, and 13.5% are wasted. Spirulina production in Zambia commenced in 2015 on a small scale, and validated its producibility in ponds. Locally produced spirulina is not currently available in shops; however, imported spirulina in liquid and tablet form is available in shops in Zambia.

## Materials and methods

### Study area

This study was conducted in the Kalaba camp of the Mansa district and the Njipi camp of the Samfya district in the Luapala province of Zambia, where the growth of 56% of children is stunted [22]. Maize-based porridge is a common complementary food used in this area of Zambia. At baseline survey, the children in these camps showed poorer nutrition status compared to the national average in both height-for-age z-scores (HAZ) (-1.68) and weight-for-age z-scores (WAZ) (-1.06), while the average education status of the mothers was also lower than the national average. In the two selected camps, 40% of the children showed stunted growth, which is a relatively better proportion than the province average. Although most children were breastfed exclusively according to the World Health Organization (WHO) guideline of six months, the average total length of breastfeeding period was 10.9 months, which is less than half of the two-year breastfeeding period recommended by the WHO.

### Study design

**Fig 1:**
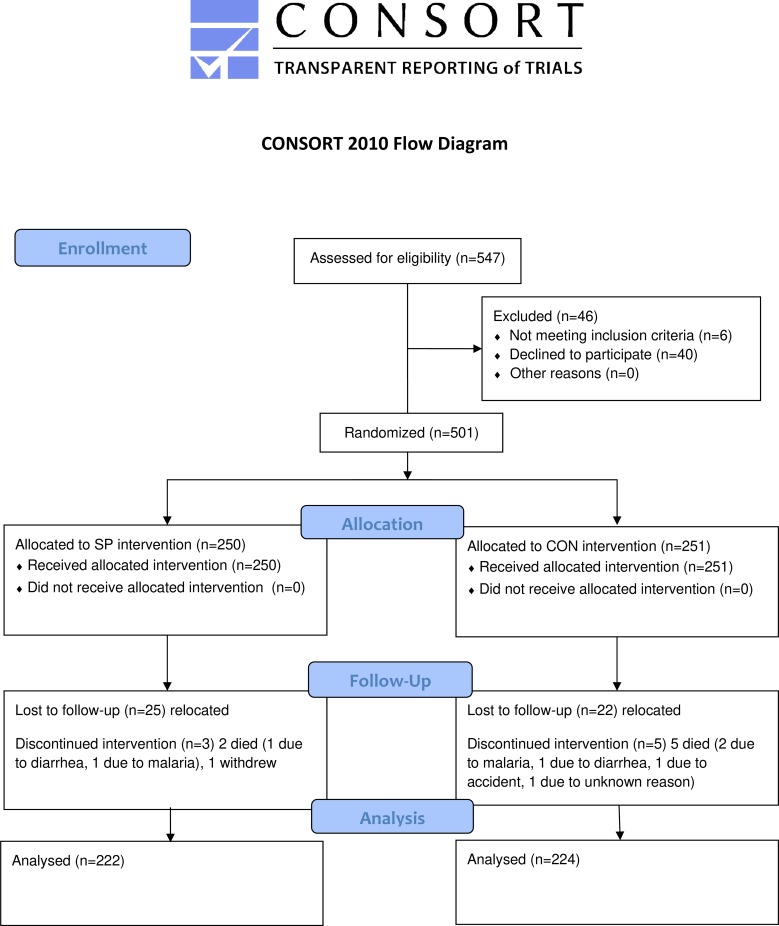
Flow chart of study participants. SP, maize-soya based control porridge plus the multiple micronutrient Spirulina; CON, maize-soya based control porridge supplementation.

This study was conducted from April 2015 to April 2016 in the form of an open-labeled randomized control trial (clinicaltrials.gov registration#: NCT03523182) that included a spirulina-fed treatment (SP) group and a control (CON) group. Initially, 547 infants under the age of 12 months from the study area were screened using the list of birth records from the local health center and invited to participate in the trial from March 6^th^, 2015 to March 31^st^, 2015. Inclusion criteria included: 1) between 6 and 18 months of age, 2) a singleton birth child, 3) residence in the study area, and 4) informed consent from at least one caregiver. Exclusion criteria were: 1) presence of severe illness warranting hospitalization on the enrolment session day, and 2) enrolment in any other clinical trial. Trained local health workers contacted all potential participants, and the infants and mothers were invited to an enrollment session. Mothers of 501 eligible infants agreed to participate in the study (Fig 1). The trial registration on clinicaltrials.gov was delayed due to an oversight, and the authors confirm that all ongoing and related trials for this intervention are registered.

In April 2015, identification numbers were assigned to infants enrolled in the study, and each infant was randomly assigned to either the SP group (n = 250) or the CON group (n = 251); randomization was achieved by generating a random allocation sequence using the STATA14 software and the runiform command (StataCorp LLC, College Station, TX, USA). A placebo group, which would have received no complementary foods, was not formulated due to ethical concerns regarding withholding a standard porridge from infants who were assessed as malnourished (stunted or underweight) at baseline.

The primary outcome was the HAZ as an indicator for growth; secondary outcomes included the WAZ, morbidity, and motor development. Additionally, we collected information on compliance and dietary habits of participating infants. The follow up survey was conducted from April 16^th^, 2016 to April 27^th^, 2016.

To assess HAZ and WAZ, height and weight of the infants were measured by experienced field workers following the World Health Organization guideline [24] at baseline (April 2015) and at endline (April 2016); these measurements were later transformed to standardized scores using the WHO Multicentre Growth Standards [25]. Weight for children was taken using a digital weighting balance (SECA Portable flat scale 803, USA), and all weight were recorded to the nearest 0.1 kg. To check the accuracy of the scale, a known weight of 1 kg was used. Height was measured using a stadiometer (SECA Portable stadiometer 217, USA) equipped with a headpiece for mothers and fathers. Length was measured using the length board (SECA Portable infantometer 417, USA) for children below 24 months and those with height less than 87 cm. All height and lengths were recorded to the nearest 0.1 cm.

We evaluated five morbidity indicators during the 12 months of the study period: severe respiratory infection (probable pneumonia), cough, severe high fever (probable malaria), and any other type of fever as reported by the mother. Severe respiratory infection was defined as cough accompanied by short and rapid breathing and difficulty in breathing [26]. Severe high fever was defined based on the following clinical signs: fever with rash on the child’s body, fever with chills, shaking, nausea, or alternating high and low body temperature [27]. Data on these morbidity indicators were collected monthly by trained local health workers who queried caregivers of the children about relevant clinical signs. The caregivers were also asked to recall whether they had fed the porridge (control or spirulina-fortified, depending on the group) to the child in the last seven days; caregiver answers to this question were used to evaluate compliance.

After the study protocol approval, we decided to assess the ability of the infant to walk without assistance as a function of motor development [28, 29], and this indicator was evaluated, following the protocol of the Malawi Development Assessment Tool [30] at baseline, six months, and endline by research assistants who visited the participants’ homes.

Data on household socio-economic status (SES) and dietary habits were collected to check if the randomization was done appropriately, and to control for confounders in the subsequent analysis. SES parameters, such as parental demographic information and household economic activity, were assessed at baseline, while dietary habits were monitored quarterly during the study period. In addition, data on the food items that were fed to the child within the prior one week were collected by the trained assistant using the questionnaire-based interview during home visits at baseline and endline to monitor changes in eating behavior. The mother or caregiver was asked to describe the food or drink that the child was given starting with the previous day (7th day) to the 1st day. Following the FAO guideline, all the food or drink purchased and consumed outside the home was excluded [31]. Following an earlier study in Africa [32], food items were classified into seven categories: starchy staples, legumes, dairy, meat/poultry/fish/eggs, vitamin A-rich fruit and vegetables, other fruits/vegetables, and oil/fat/butter. Based on the collected data, a dietary diversity score of 0–7 was calculated for each child. The score represented the number of food categories that were consumed during more than half of the week by the child.

Sample size calculation was performed based on the information from our previous study, which showed that the mean HAZ among children under the age of five in Zambia in 2014 was -2.26 with a standard deviation of 0.86 [19]. We assumed the effect size of spirulina supplementation compared with control supplementation would be 0.23 (10% increase compared to the mean, or equivalent to a “small” to “medium” effect size in Cohen’s d) in the outcome. To detect this effect with a power of 0.80 and a two-sided significance level of 0.05, and with an equal division between SP and CON groups, this would require a sample size of 438. Allowing for a 10% attrition rate, the target sample size at baseline was calculated to be 487.

### Spirulina supplementation

Spirulina was produced in the USA by DIC LIFETEC Co. Ltd., Japan. We used 10 g per day of spirulina powder with a mealie meal and soya flour porridge blend. A mealie meal is made from locally available maize and has an extraction rate of about 90 per cent. Table 1 shows the details of monthly distribution of the porridge blend to each group. The blending was done by assistants and the two blends were different in color and taste. Ideally, we provided a matching placebo for the control blend to mask the treatment status. However, blinding was not possible because the deep color and flavor of spirulina did not allow us to mask the treatment. Assistants delivered the cold porridge blend to each home every month, and spirulina, in the form of cold powder, was added to the porridge blend for the treatment group only. Assistants trained the care givers in how to prepare the porridge, and advised the care giver that the participating infants should consume the porridge three times per day. First, water was boiled and then 70 g blend paste was stirred into the boiling water and cooked for 20–30 minutes. After cooling the porridge, it was then fed to the child while still hot or warm. During home visits, assistants monitored this process as often as was possible to ensure compliance. Compliance was measured by the number of days that the infants had eaten the distributed porridge during the seven days preceding the assistant’s home visit. Supplementation was performed for 12 months.

**Table 1:** The amount of the porridge blend distributed by group.

	Mealie Meal	Sugar	Salt	Soya	Spirulina
Control Porridge	4 kg	0.8 kg	0.1 kg	1 kg	
(for CON group)					
Spirulina Porridge	4 kg	0.8 kg	0.1 kg	1 kg	0.3 kg
(for SP group)					

CON, control; SP, spirulina porridge

The intervention was designed to deliver beta-carotene (1800 μg retinol equivalent (RE)), and iron (8.3 mg) using 10 g of spirulina per day. Spirulina-fortified porridge also includes vitamin B1, B2, B3, B6, B12, vitamin E, vitamin K, calcium, phosphorus, niacin, sodium, potassium, magnesium, zinc, and copper. The detailed macronutrient and micronutrient composition of the porridge in the two groups was compiled based primarily on food composition data from the Zambia Food Composition Tables published by the Zambian National Food and Nutrition Commission [33], adding data from standard tables of food composition in Japan and FAO, and Siva et al. [12, 34, 35], and is shown in Table 2.

**Table 2:** Nutrient composition of the soya and spirulina supplements used in this study.

Vitamin/ mineral/ macro nutrients	Spirulina (10 g)	Soy (40 g): Control group	Spirulina 10 g + Soy 40 g: Treatment group
β-carotene (μg RE)	1800	22	1822
Vitamin B1 (mg)	0.48	0.284	0.764
Vitamin B2 (mg)	0.39	0.1	0.49
Vitamin B3 (mg)	3.9	0.8	4.7
Vitamin B6 (mg)	0.09	0.184	0.274
Vitamin E (mg)	1.06	9.12	10.18
Vitamin K (μg)	222	13.6	235.6
Folic acid (μg)	7.3	88	95.3
Calcium (mg)	7.05	73.2	80.25
Phosphorus (mg)	92.1	216.4	308.5
Iron (mg)	8.33	2.44	10.77
Sodium (mg)	21	0.4	21.4
Potassium (mg)	152	720	872
Magnesium (mg)	27.8	92	119.8
Zinc (mg)	0.104	1.8	1.904
Copper (mg)	0.026	0.388	0.414
Calories	38.6	162	200.6
Protein (g)	69.4	13.48	82.88
Total fat (g)	0.82	7.16	7.98
Total carbohydrate (g)	1.27	13.56	14.83

### Ethical statement

The study protocol was approved by the Biomedical Research Ethics Committee of the University of Zambia on March 5th, 2015 (ethical reference number: IRB00001131 of IORG0000774). This study ensured voluntary participation and participant confidentiality throughout the study. Written informed consent was obtained from the parents of participating infants.

### Statistical analysis

Data were entered into an electronic database by trained assistants and analyzed using the STATA14 software (StataCorp LLC, College Station, TX, USA).

To identify the effects of spirulina intake on infant growth and morbidity, we followed the difference in differences approach. Namely, our strategy was to compare changes in the children’s growth and morbidity in the households who received spirulina over the study period to that in households that did not receive spirulina. To determine the effects of spirulina intake, we pooled the observations from both baseline and endline surveys, and performed a regression of the outcome on the interaction term between the treatment status and the binary variable; this binary variable takes the value of 1 if observation was collected in April 2016, and 0 if observation was collected in April 2015. We denoted the outcome of a child *i* in period *t* as ***y***_***it***_ and the child’s status in terms of whether he/she received spirulina as ***Treatment***_***i***_ (i.e. 1 if an infant is in SP the group, 0 otherwise), and whether his or her status was collected at endline as ***Endline***_***t***_. The regression model was as follows:

$$y_{it}=\alpha +\beta Endline_{t}+\gamma Endline_{t}*Treatment_{i}+\lambda_{i}+\epsilon_{it}$$ (1)

***β*** represents the common change in the outcome of the infants in the SP and CON groups over the 12-month intervention period. Coefficient of our interest is ***γ*,** which describes the treatment effects; we would expect ***γ*** to be positive if the provision of spirulina increases the infant’s weight and height gain.

Contrary to the growth and morbidity indicators, data on the motor development parameters were available only at endline, because a sub-group of participating infants were initially below 12 months of age at baseline, and it was too early to assess these indictors. In those cases, we compared the outcome in the two groups at endline by using Eq (2).

$$y_{i}=\mu +\delta Treatment_{i}+\xi X_{i}+\eta_{i}$$ (2)

***δ*** represents the treatment effects, and includes several baseline characteristics of infants, their mothers, and the households; ***X***_***i***_ allows us to isolate the treatment effects from other confounding factors. We expect ***δ*** to be positive if the spirulina supplementation increases the probability of walking at 12, 13, 14, and 15 months of age in the SP group, compared with that in the CON group.

The Ordinary Least Square (OLS) linear regression was used to solve Eq (1) regardless of whether the outcome was a dichotomous variable or a continuous variable; this was done for ease of interpretation of marginal effects and inclusion of the extensive set of covariates, including individual fixed effects. Standard errors were clustered at the individual level to address intra-cluster correlation of standard error. Eq (2) was evaluated using the probit regression model. All observations available at the endline point were included to conduct intention-to-treat treatment analysis using the full analysis set.

In the following sections, the balance between the SP group and CON group at the start of the study was verified to show the validity of the randomization procedure. After the balance check, Eqs (1) and (2) were evaluated by OLS regression to assess the intention-to-treat effects of spirulina supplementation on the linear growth, morbidity, and motor milestone acquisition of the participating infants.

## Results

### Participant flow

Infant enrollment was conducted from January to March 2015. All 547 eligible infants were selected and invited for the intervention, but 46 did not enroll because the mother declined or we could not locate the potential participant’s home due to migration. Thus, we collected baseline information from 501 participants, and randomly assigned them to one of two study groups (SP or CON group). As a result, 250 participants received the spirulina treatment and 251 received the control supplementation. Seven (1.4%) of 501 infants died during the study. Five of them were observed in CON group, and two of which died due to malaria, others died due to diarrhea, accident, and an unknown reason. Two fatalities were observed in the SP group, and they died due to malaria and diarrhea. The mortality rates did not differ across the groups. Another 48 (9.6%) infants did not complete the study. Twelve children migrated out of the study site, five withdrew due to objection from the parents, and 31 withdrew due to unspecified reasons. The attrition rates were low and not statistically different between the SP and CON groups. The propensity of attrition was not systematically associated with any of the baseline characteristics of the participants (S2 Table). The final data set consisted of 446 children (SP: n = 222; CON: n = 224). All 446 observations available at the endline point were included to conduct intention-to-treat treatment analysis using the full analysis set.

### Balance check at baseline

Table 3 describes the selected 11 indicators of SES and health status in the two study groups, and depicts the differences between groups at baseline.

**Table 3:** Baseline characteristics of the participants by intervention group.

	SP	CON	p-value
	(n = 222)	(n = 224)	
*Child characteristics*			
Height (cm)	68.4±8.6	69.3±5.4	0.21^a^
Weight at baseline (kg)	8.3±4.7	8.3±4.4	0.93 ^a^
Child had fever during last 4 weeks (%)	76.3	75.2	0.80 ^b^
Child had diarrhea during last 4 weeks (%)	44.1	42.6	0.75 ^b^
Age (months)	10.6±3.7	11.2±3.8	0.11 ^a^
Dietary diversity score (0–7)	3.8±1.9	3.8±2.0	0.74 ^a^
*Maternal characteristics*			
Maternal age (year)	27.9±6.5	27.5±7.4	0.54 ^a^
Maternal height (cm)	152.0±14.2	153.7±10.0	0.13 ^a^
Maternal weight (kg)	49.3±7.2	49.7±8.2	0.59 ^a^
Maternal education (years)	6.2±4.7	6.0±4.3	0.52 ^a^
*Household characteristics*			
Number of children under the age 5	2.2±0.9	2.2±1.0	0.50 ^a^
ln (value of household asset in Zambian Kwacha)	5.2±2.0	5.2±2.0	0.97 ^a^
Households which have access to electricity (%)	0.9	0.9	0.99 ^b^

Note: CON, control porridge group; SP, spirulina porridge group. CON group received porridge with soya. SP group received the same distribution plus spirulina. We take the log of “value of household asset in Zambian Kwacha” for the ease of interpretation of regression coefficient.

Groups were compared using the t-test^a^ and chi-square test^b^, and the p-value is presented in the last column of the table. The values in the first and second columns show Mean±SD.

None of the 11 characteristics of the infants, mothers, or of the households were significantly different across the two groups. Overall, the results from the balance test suggested that the baseline characteristics of the infants in the two groups were similar. Because infants in the two groups were similar except for spirulina treatment, the observed differences in the study outcomes could be attributed to the intake of nutrients from spirulina.

### Effects on physical growth

**Fig 2:**
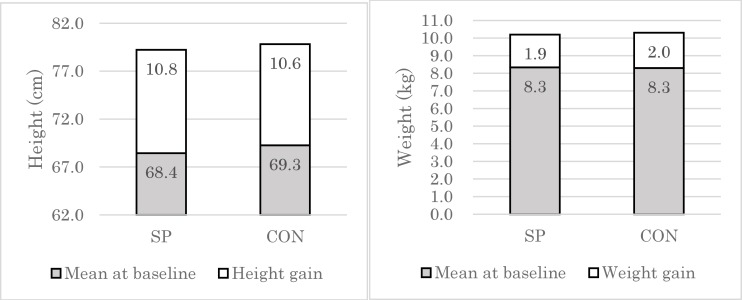
Height and weight gain of the infants over 12 months by group. Mean height in cm (left) and mean weight in kg (right) of participant infants at baseline and endline is represented in dark and light color, respectively, and graphed by group. SP, maize-soya based control porridge plus the multiple micronutrient spirulina; CON, maize-soya based control porridge supplementation.

At baseline, infants in the two groups did not differ in terms of height and weight (Table 3). During the 12 months of the study period, infants in the SP group gained height (10.8 cm) and weight (1.9 kg), but the infants who received the control porridge gained similar height and weight (10.6 cm, 2.0 kg, respectively) (Fig 2).

In order to control for the time-invariant observed and unobserved differences among infants at baseline, we estimated Eq (1) by linear regression. Table 4 shows the effects of spirulina provision on height, weight, HAZ, and WAZ. Infants in both the SP and CON groups gained height by 11.3 cm (95% CI: 9.17, 13.36; P < 0.01) on average. However, the insignificant coefficient of the interaction term (-0.13; 95% CI: -1.23, 0.98; P > 0.10) suggests that the change in infant height was not statistically different between infants in the SP and CON groups even after controlling for observed and unobserved time-invariant individual characteristics. This finding was consistent even when we assessed the effects on weight, HAZ, and WAZ. In summary, the results suggest that, in the present study, spirulina supplementation did not significantly improve infant growth indicators as compared to control supplementation.

**Table 4:** The effects of spirulina supplementation on infant growth.

Estimated association with following explanatory variables	Height	Weight	Height for Age Z-score (HAZ)	Weight for Age Z-score (WAZ)
1 if endline	11.27***	2.90***	3.58***	2.26***
	(9.17, 13.36)	(2.08, 3.72)	(2.89, 4.27)	(1.77, 2.74)
[1 if endline]*treatment	-0.42	-0.11	-0.08	-0.12
	(-1.52, 0.69)	(-0.34, 0.12)	(-0.30, 0.14)	(-0.30, 0.06)

Note: Values are estimated regression coefficients with 95% CIs. All specifications include individual fixed effects to control for time invariant individual characteristics. 95% confidence intervals are in parenthesis.

*** stands for significance at 1% level

** at 5% level, and

* 10% level from t-test.

### Effects on morbidity

**Fig 3:**
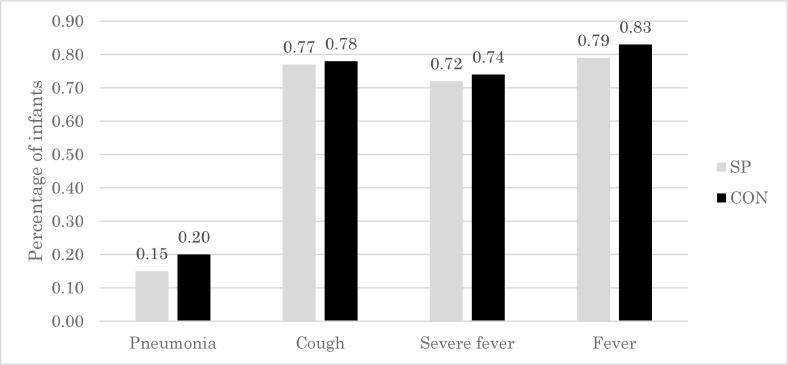
Percentage of infants with pneumonia, cough, severe fever, and fever over the 12-month study period, by group. SP, maize-soya based control porridge plus the multiple micronutrient spirulina, which provides vitamins and minerals; CON, maize-soya based control porridge supplementation.

At baseline survey, morbidity rates did not differ between the two groups except for cough-related infection, which was higher in the SP group (Table 1). After 12 months of intervention, the incidence of probable pneumonia in the SP group (0.15) was lower than that in the CON group (0.20) (Fig 3). The incidence of cough, severe high fever, and any other fever was also lower in the SP group (Fig 3).

Table 5 shows the effects of spirulina supplementation on the incidence of probable pneumonia, cough, severe high fever, and fever. The results suggest that, after controlling for time-invariant characteristics including the incidence of cough at baseline, spirulina supplementation reduced the incidence of cough by 11% (95% CI: -0.23, -0.00; P < 0.05), compared to control supplementation. Spirulina supplementation also showed a trend of reducing the incidence of pneumonia, severe fever, and fever compared to control supplementation, although the effect was not significant. In summary, these results suggest that spirulina supplementation prophylactically prevented upper respiratory infection morbidity in the participating infants.

**Table 5:** The effects of spirulina intake on infant morbidity.

Estimated association with following explanatory variables	1 if a child suffered from …. during last 12 months			
	Pneumonia	Cough	Severe high fever (Malaria)	Fever
1 if endline	-0.22*	-0.03	-0.02	-0.05
	(-0.46, 0.02)	(-0.31, 0.24)	(-0.24, 0.19)	(-0.30, 0.21)
[1 if endline]*treatment	-0.07	-0.11**	-0.03	-0.09
	(-0.17, 0.04)	(-0.23, -0.00)	(-0.13, 0.06)	(-0.19, 0.02)

Note: Values are estimated regression coefficients with 95% CIs. All specifications include individual fixed effects, and dummy variables for child age in months. 95% confidence intervals are in parentheses.

*** stands for significance at 1% level

** at 5% level, and

* 10% level from t-test.

### Effects on motor milestone acquisition

**Fig 4:**
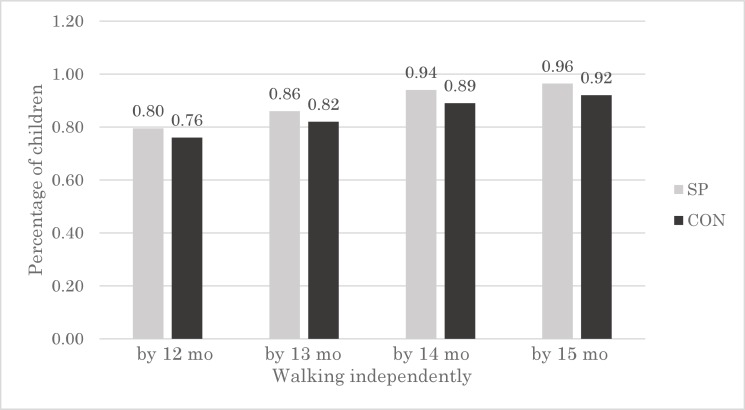
Percentage of children walking independently by 12, 13, 14, and 15 months, by group. SP, maize-soya based control porridge plus the multiple micronutrient spirulina, which provides vitamins and minerals; CON, maize-soya based control porridge supplementation.

To explore whether micronutrients from spirulina may reduce the time to acquire motor milestones among infants, we compared the proportion of children who were able to walk without assistance at 12, 13, 14, and 15 months between the two groups. The probability of walking independently at 12 months was higher (by 4 percentage points) in the SP group (0.80) compared with that in the CON group (0.76) (Fig 4). This difference between groups was observed even when we assessed motor milestone acquisition at 15 months.

To isolate treatment effects from confounders, linear regression was performed (Table 6). The results show that the probability of children being able to walk by 12 months and 13 months did not differ between the two groups. This observation is possibly because the mean age of infants at baseline was 11 months (Table 2) and thus the duration of spirulina supplementation was too short to change their motor development at 12 months and 13 months. In contrast, we observed differences in the motor development of the SP group at 14 months and 15 months (Table 2). The infants in the SP group were nine percentage points and eight percentage points more likely to walk unassisted at 14 months (95% CI: 0.02, 0.15; P < 0.01; OR = 2.55; 95% CI: 1.26, 5.14) and at 15 months (95% CI: 0.02, 0.14; P < 0.01; OR = 3.12; 95% CI: 1.37, 7.07), respectively, compared to those in the CON group. In summary, these results suggest that the fortification of porridge using spirulina improved the motor development of infants.

**Table 6:** The effects of spirulina supplementation on the probability that a child is able to walk independently by 12–15 months.

Outcome: The probability that a child can walk independently by ….	12 mo	13 mo	14 mo	15 mo
	0.07	0.08*	0.09***	0.08***
	(-0.02, 0.16)	(-0.00, 0.16)	(0.02, 0.15)	(0.02, 0.14)

Note: Estimated by probit model. Values are estimated regression coefficients with 95% CIs. Each specification limits the sample only to children whose age is above the relevant age. For example, column 1 includes children who are 13 months old or older at the endline point. All specifications control for individual characteristics: age in months, gender, 1 if child had suffered from malaria, measles before baseline point, and mothers' characteristics: mother's age, height, and weight.

*** significance at 1% level

** at 5% level, and

* 10% level from t-test.

### Difference in food intake other than distributed porridge

To test whether the treatment (control) group changed their pattern of food consumption other than the distributed porridge, compared to the control (treatment) group, we compared the change in the dietary diversity score between the two groups (Table 7). The results suggest that the two groups did not differ in terms of quality of food intake other than the distributed porridge with or without spirulina. In summary, observed differences in child upper respiratory infection morbidity and time to walk unassisted are unlikely to be produced spuriously by intake of foods other than spirulina.

**Table 7:** The effects of spirulina supplementation on infant dietary diversity.

Estimated association with following explanatory variables	Dietary diversity score
1 if endline	1.11***
	(0.84, 1.38)
[1 if endline]*treatment	0.25
	(-0.13, 0.63)

Note: Values are estimated regression coefficients with 95% CIs. Dietary diversity score is a value between 0 and 7, with a higher value indicating greater diversity. All specifications include individual fixed effects to control for time invariant individual characteristics. 95% confidence intervals are in parentheses.

*** stands for significance at 1% level

** at 5% level, and

* 10% level from t-test.

## Discussion

Our study showed similar increase in linear growth between infants that received spirulina-fortified porridge supplementation and those that received control porridge supplementation. These results are contrary to a similar study in Zambia [19], which reported that the daily intake of fortified porridge using spirulina was associated with greater height gain compared to a control group that received no porridge supplement. However, the control group in the present study also received a porridge made with a soybean-based powder; only the addition of spirulina was excluded, and the amount of protein and energy supplied to the SP group and the CON group was almost equivalent. Hence, infants in the CON group in the present study may have shown a better increase in linear growth than children in the previous study. Although spirulina supplementation provides MMN to infants and micronutrient, e.g., zinc, deficiency is known to negatively affect growth of children [36, 37], several randomized trials have shown that MMN supplementation has little effect on growth. Our results are consistent with the IRIS clinical studies [38–41], and other studies in Africa [42–43] and in Cambodia [44] which have shown that MMN supplementation was not associated with infant growth. These findings suggest that supplementation with MMN alone may not improve the growth of infants in some populations in resource-poor settings.

Furthermore, no effects of spirulina on linear growth in this study may have been due to an intervention length of only 12 months. A previous study in Mexico provided MMN supplements to infants from three months to 24 months of age, and found that MMN supplements increased the height of children who consumed them regularly [45]. Hence, future analysis is needed to confirm whether spirulina supplementation for longer than 12 months improves infant growth. A third reason for the lack of positive effects on child growth in the present study could be related to the age of infants at baseline. In a study conducted in India, children whose mean age was 23 months (compared with 11 months in the present study) received MMN-fortified milk for 12 months, and showed a larger height and weight gain than did children who received control milk [46]. Therefore, it is possible that if our intervention had targeted an older age group of children, positive effects on child growth might have been obtained.

Our study also showed similar body weight gain between the SP group and CON group, suggesting spirulina intake had little effect on body weight of malnourished infants. This result is consistent with the study in Burkina Faso that used spirulina platensis as a micronutrient supplement for malnourished children [17], and the other types of multiple micronutrient supplementation [47] to treat child malnutrition in developing countries. Some recent studies in Iran reported that 1–2 g/day spirulina supplementation for 12 weeks reduced body weight and BMI among obese healthy adults [48, 49]. This is probably because of the hypolipidemic effects of spirulina reported in previous clinical studies [50–52]. The lack of impact on body weight or WAZ among malnourished infants in the present study may appear contradicting to their findings, but such discrepancies are likely to be attributed to differences in the obesity level of the participants.

Our data also showed that infants in the SP group were 11 percentage points less likely to develop a cough than were children in the CON group. These findings suggest positive effects of spirulina fortification for reducing respiratory infection. Data is varied from previous studies related to the effectiveness of micronutrient supplementation for reducing infant morbidity. In a study conducted in Ghana, no difference was found in the prevalence of illness, diarrhea, and pneumonia between infants who received the fortified complementary food and the infants in the placebo group [22]. Another study, conducted in South Africa, showed that when compared to vitamin A supplementation only, zinc supplementation with or without other micronutrients did not reduce the incidence of diarrhea and respiratory infection [53]. However, our results are consistent with a study conducted in India, which showed that milk fortified with zinc, iron, and other micronutrients reduced the incidence of severe illness, diarrhea, and acute respiratory infection compared to control milk [54]. These findings suggest that home-fortification of complementary foods with MMN reduces the incidence of morbidity in some infant populations.

It is interesting to note that the effects of spirulina on the incidence of respiratory infection may be due to simultaneous supplementation with zinc and iron. In Bangladesh, infants received iron alone, zinc alone, or iron with zinc supplementation, and the results showed that supplementation with iron alone or with zinc alone had no effect on the incidence of diarrhea and acute lower respiratory infection. However, when iron and zinc were given together, effects on disease incidence were observed [55]. The nutrient-rich nature of spirulina is optimally suited to reduce infant morbidity; however, as it contains MMN, it is difficult to determine the role of each micronutrient in improving infant health. Thus, it is important for future studies to explore the mechanism through which spirulina intake may reduce the incidence of morbidity.

In the present study, spirulina supplementation reduced the time to reach the measured motor development milestone (walking independently). This finding is consistent with a study conducted in Ghana, which showed that infants who received three types of MMN-fortified complementary foods had higher chances of being able to walk independently by 12 months than did infants who received a placebo [42]. Similarly, in a study conducted in Zanzibar, infants who received iron supplementation began to walk at an earlier age than did those who did not receive iron supplementation [56]. A randomized trial in Bangladesh found that iron and zinc supplementation with other micronutrients had beneficial effects on time to walk unassisted [57]. These findings suggest that it is important to provide MMN to infants in poor nutritional settings, even if the effects of MMN on physical growth are not directly apparent.

Existing evidence suggests that physical growth and iron deficiency predict infants’ attainment of walking independently among infants who are poorly nourished [58, 59]. Given that spirulina supplementation did not improve the growth of treated infants compared with control infants in the present study, the effects of spirulina may be mediated by improvements in iron absorption. Although the nature of the data in the current study did not allow for examination of changes in anemic status of participating infants, it would be interesting to explore the effects of spirulina supplementation on iron deficiency in future studies.

It is also possible that the effects of treatment on child motor development might be mediated by the protective effects of the spirulina on respiratory infection. A previous study has shown an association between lung function and development in children [60]. Given that our spirulina supplementation reduced the incidence of respiratory disease in the present study, it may indirectly increase the energy intake and decrease the energy expenditure and thus improve the motor development. It could well be possible that the causality goes the other way, i.e. improved motor development could reduce the respiratory infection. Although our data set does not allow us to isolate one effect from the other, it would be very informative to uncover how these two factors interact each other in future studies.

### Limitations

Our study has several limitations. The first limitation of our design was that it was not possible to conduct the study in a blinded fashion, and the mothers and the assistants who delivered the porridge were aware of the treatment allocation details. Hence, the awareness of being treated may have influenced the caregivers’ attention to children in the SP group, an effect known as the Hawthorne effect. However, the compliance rates between the two groups in the present study did not differ, and the dietary diversity of the infants changed similarly over time in the two groups. We further tested the sensitivity of our results by including the dietary diversity score in the set of explanatory variables in Eqs (1) and (2), and the results were not sensitive to this change in the specification (S1 Table). Thus, it is unlikely that the study results were significantly influenced by the Hawthorne effect.

Another concern derived from non-masking was that the distributed porridge fortified with spirulina may have been shared by other household members because the mothers knew it was nutritious. To address this concern, we would ideally have recorded the dietary intake of all household members, but this would be too costly to implement in practice. As suggestive evidence, we compared the change in weight and morbidity among mothers in the two groups. If porridge distributed to the treatment group was shared with the mother, we expect that mothers in the treatment group would have greater weight and reduced morbidity. Inconsistent with this concern, we found no difference in these parameters between the groups. Hence, it is unlikely that mothers in the SP group would have eaten a portion of distributed porridge. However, it is possible that the mothers may have shared the porridge with siblings of the participating infants, thus reducing the amount of porridge received by the target infants. Nevertheless, if this sharing did occur, it would most likely occur in the SP group rather than in the CON group. This would have resulted in a lower intake of porridge among infants in the SP group compared to those in the control group; if that were the case, the difference between the two groups is likely to have been underestimated, and our estimates of the effects size may have been lower than the true effects.

The second limitation of our study was that the intervention lasted only 12 months, and the timing of data collection allowed us to only examine the acute effects of spirulina supplementation. As discussed in the previous section, our data suggests that spirulina supplementation improved motor milestone acquisition among infants compared with control supplementation. It thus follows that spirulina intake may potentially have positive and sustained impact on intelligence, working memory, and other cognitive functions later in a child’s life. In a study conducted in Peru, infants initially aged six months received micronutrient supplementation for six months, and were observed to have reduced anemia prevalence compared with those who received iron supplementation alone, although no differences in cognitive ability at the age of three were recorded between the two groups [61]. In the present study, we could not assess whether spirulina supplementation had any long-lasting positive effects on the cognitive ability of participating infants.

Third, we have assessed, and reported the effectiveness of spirulina supplementation at 12 months, because the motor development measurement was only available at 12 months; moreover, this meant the baseline and endline surveys were conducted in the same month of the year, allowing us to control for seasonality in agricultural productivity and morbidity, but information lacking between these two surveys may be another limitation. To validate the effectiveness of spirulina supplementation, analysis using a middle line survey at 6 month is presented in the S3 Table, and the results are consistent with findings at 12 months. This further supports that spirulina supplementation consistently reduced cough morbidity during the study period.

Lastly, the attrition rate (9.6%) is lower than comparable trials in developing countries [38, 46], but it may also be a limitation. Such attrition is, however, unlikely to have introduced bias in the results of the study. Our analysis shows that the baseline characteristics of the infants who dropped out were not statistically different from the rest of the infants (S2 Table). This suggests that attrition occurred as-if-randomly, and is unlikely to change the distribution of the baseline sample. Therefore, infants in the SP group and CON group in the full analysis set remains comparable. It is important to note that the low attrition rate and lack of systematic differences in the baseline characteristics between the SP and CON groups suggest that our estimates may have high generalizability to the study population.

In summary, based on the findings of the present study, we conclude that fortification of complementary infant food with spirulina had beneficial effects on infant upper respiratory infection morbidity and motor development. Spirulina may thus be a cost-effective home-fortification agent to improve infant health in resource-poor countries.

## Supporting information

S1 Checklist CONSORT checklist. (DOC)

S1 Table Sensitivity analysis using dietary diversity score as a control variable. (DOCX)

S2 Table Correlate of attrition with household characteristics at baseline. (DOCX)

S3 Table A. The effects of spirulina supplementation on infant growth at 6 month. B. The effects of spirulina intake on infant morbidity at 6 month.
(DOCX)

S1 Protocol Trial protocol. Reprinted from Promoting Spirulina Production and Utilization in Luapula Province of Zambia Research Protocol under a CC BY license, with permission from Programme Against Malnutrition, original copyright 2014. (PDF)
