# Supplementary material for: Multiple micronutrient supplementation using spirulina platensis and infant growth, morbidity, and motor development: Evidence from a randomized trial in Zambia
Source: PLoS One. 2019 Feb 13;14(2):e0211693. doi: 10.1371/journal.pone.0211693 (PMC6373937; doi:10.1371/journal.pone.0211693)
Supplement: S1 Table — (DOCX) [file pone.0211693.s002.docx]

**S1 Table. Sensitivity analysis using dietary diversity score as a control variable**

**The effects of spirulina supplementation on infant growth.**

| Estimated association with  following explanatory variables | Height | Weight | Height for Age Z-score (HAZ) | Weight for Age Z-score (WAZ) |
| --- | --- | --- | --- | --- |
| 1 if endline | 11.32*** | 2.94*** | 3.55*** | 2.30*** |
|  | (9.17 , 13.47) | (2.10 , 3.77) | (2.87 , 4.23) | (1.81 , 2.79) |
| [1 if endline]*treatment | -0.49 | -0.11 | -0.11 | -0.12 |
|  | (-1.61 , 0.64) | (-0.35 , 0.12) | (-0.34 , 0.11) | (-0.30 , 0.06) |

Note: Values are estimated regression coefficients with 95% CIs. All specifications include dietary diversity score, dummy variables for child age in months, and individual fixed effects to control for time invariant individual characteristics. 95% confidence intervals are in parenthesis. *** stands for significance at 1% level, ** at 5% level, and * 10% level.

**The effects of spirulina intake on infant morbidity.**

| Estimated association with  following explanatory  variables | 1 if a child suffered from …. during last 12 months | | | |
| --- | --- | --- | --- | --- |
|  | Pneumonia | Cough | Severe high fever (Malaria) | Fever |
|  |  |  |  |  |
| 1 if endline | -0.18 | -0.01 | -0.14 | -0.01 |
|  | (-0.43 , 0.06) | (-0.29 , 0.27) | (-0.40 , 0.12) | (-0.26 , 0.25) |
| [1 if endline]*treatment | -0.07 | -0.13** | -0.04 | -0.08 |
|  | (-0.17 , 0.04) | (-0.24 , -0.01) | (-0.17 , 0.08) | (-0.18 , 0.03) |

Note: Values are estimated regression coefficients with 95% CIs. All specifications include dietary diversity score, dummy variables for child age in months, and individual fixed effects to control for time invariant individual characteristics. 95% confidence intervals are in parentheses. *** stands for significance at 1% level, ** at 5% level, and * 10% level.

**The effects of spirulina supplementation on the probability that a child is able to walk independently by 12–15 months**

| Outcome: The probability that a child can walk independently by …. | 12 mo | 13 mo | 14 mo | 15 mo |
| --- | --- | --- | --- | --- |
| 1 if treatment | 0.07 | 0.08** | 0.09*** | 0.08*** |
|  | (-0.02 , 0.16) | (0.00 , 0.16) | (0.02 , 0.15) | (0.03 , 0.14) |

Note: Estimated by probit model. Values are estimated regression coefficients with 95% CIs. Each specification limits the sample only to children whose age is above the relevant age. For example, column 1 includes children who are 13 months old or older at endline point. All specifications control for individual characteristics: dietary diversity score, age in months, gender, 1 if child had suffered from malaria, measles before baseline point, and mothers' characteristics: mother's age, height, and weight. *** significance at 1% level, ** at 5% level, and * 10% level.
