# Supplementary material for: Multiple micronutrient supplementation using spirulina platensis and infant growth, morbidity, and motor development: Evidence from a randomized trial in Zambia
Source: PLoS One. 2019 Feb 13;14(2):e0211693. doi: 10.1371/journal.pone.0211693 (PMC6373937; doi:10.1371/journal.pone.0211693)
Supplement: S2 Table — (DOCX) [file pone.0211693.s003.docx]

**S2 Table. Correlate of attrition with household characteristics at baseline**

| Outcome | 1 if attrited between baseline and endline | |
| --- | --- | --- |
| Age of child in months | 0.00 | (-0.01 - 0.01) |
| 1 if female | -0.01 | (-0.07 - 0.05) |
| Number of household member | 0.01 | (-0.01 - 0.03) |
| Number of under 5 member | -0.03 | (-0.06 - 0.01) |
| ln(household consumption) | -0.01 | (-0.04 - 0.02) |
| ln(household expenditure) | 0.02 | (-0.01 - 0.04) |
| 1 if lives in Mansa | 0.04 | (-0.02 - 0.10) |
| 1 if received any government assistance in last 12 months | 0.02 | (-0.12 - 0.15) |
| 1 if with diarrhea | -0.00 | (-0.06 - 0.05) |
| 1 if with fever | -0.03 | (-0.10 - 0.03) |
| 1 if with cough | 0.03 | (-0.04 - 0.10) |
| Mother's age | -0.00 | (-0.01 - 0.00) |
| Mother's years of education | 0.00 | (-0.01 - 0.01) |
| 1 if in tratment group | 0.00 | (-0.06 - 0.06) |

Note: Values are estimated regression coefficients with 95% Cis in parenthesis.
 *** stands for significance at 1% level, ** at 5% level, and * 10% level.
