# Supplementary material for: Multiple micronutrient supplementation using spirulina platensis and infant growth, morbidity, and motor development: Evidence from a randomized trial in Zambia
Source: PLoS One. 2019 Feb 13;14(2):e0211693. doi: 10.1371/journal.pone.0211693 (PMC6373937; doi:10.1371/journal.pone.0211693)
Supplement: S3 Table — A. The effects of spirulina supplementation on infant growth at 6 month. B. The effects of spirulina intake on infant morbidity at 6 month. (DOCX) [file pone.0211693.s004.docx]

**S3A Table. The effects of spirulina supplementation on infant growth at 6 month.**

| Estimated association with  following explanatory variables | Height | Weight | Height for Age Z-score (HAZ) | Weight for Age Z-score (WAZ) |
| --- | --- | --- | --- | --- |
| [1 if endline]*treatment | -0.15 | -0.09 | -0.03 | -0.16 |
|  | (-1.07, 0.78) | (-0.27, 0.09) | (-0.24, 0.18) | (-0.32, 0.00) |

Note: Values are estimated regression coefficients with 95% CIs. All specifications include individual fixed effects to control for time invariant individual characteristics. 95% confidence intervals are in parenthesis. *** stands for significance at 1% level, ** at 5% level, and * 10% level from t-test.

**S3B Table. The effects of spirulina intake on infant morbidity at 6 month.**

| Estimated association with  following explanatory  variables | 1 if a child suffered from …. during last 12 months | | | |
| --- | --- | --- | --- | --- |
|  | Pneumonia | Cough | Severe high fever (Malaria) | Fever |
| [1 if middle line]*treatment | -0.03 | -0.08** | 0.01 | -0.02 |
|  | (-0.12, 0.07) | (-0.15, -0.01) | (-0.08, 0.10) | (-0.10, 0.06) |

Note: Values are estimated regression coefficients with 95% CIs. All specifications include individual fixed effects, and dummy variables for child age in months. 95% confidence intervals are in parentheses. *** stands for significance at 1% level, ** at 5% level, and * 10% level from t-test.
