# Supplementary material for: Multiple micronutrient supplementation using spirulina platensis and infant growth, morbidity, and motor development: Evidence from a randomized trial in Zambia
Source: PLoS One. 2019 Feb 13;14(2):e0211693. doi: 10.1371/journal.pone.0211693 (PMC6373937; doi:10.1371/journal.pone.0211693)
Supplement: S1 Protocol — (PDF) [file pone.0211693.s005.pdf]

Promoting Spirulina Production and Utilization in Luapula Province of Zambia

Research Protocol

ver 1.0

By:

Maureen Chitundu, Acting Executive Director - Programme Against Malnutrition
Yumiko Ota, Deputy Programme Manager - Alliance Forum Foundation

5 March 2015

Table of Contents

1 Background and Introduction ... 1

1.1 Rationale for study ... 1

1.2 Significance ... 3

1.3 Aim ... 3

1.4 Specific objectives ... 3

1.5 Ethical Issues ... 4

2 Literature Review ... 4

3 Methodology ... 5

3.1 Project Sites ... 5

3.2 Target Population ... 5

3.3 Research Process ... 6

3.4 Duration ... 7

3.5 Assessment Criteria ... 7

3.6 Training ... 7

3.7 Detailed Ethical issues ... 7

4 Budget ... 8

5 Time Frame ... 8

References ... 10

Appendix 1: Information Sheet (English) ... 12

Appendix 2: Information Sheet (Bemba) ... 25

Appendix 3: Consent Form (English) ... 38

Appendix 4: Consent Form (Bemba) ... 40

Appendix 5: Interview Guide ... 42

Appendix 6: Body Measurement Record Format ... 43

Appendix 7: Socio-Economic Status Questionnaire ... 44

Appendix 8: Dietary Diversity Questionnaire ... 58

Appendix 8: Monthly Morbidity Questionnaire ... 67

Appendix 9: Letters of Approval ... 70

Appendix 10: Investigator CV ... 74

1 Background and Introduction

Infant and child malnutrition is one of the most serious health problems in developing countries. Malnutrition impairs a child's physical and mental development and can result in lower IQ and compromised immunity. Lancet Series (2013) reported that 45 % of children's deaths in developing countries are caused by malnutrition. According to the World Health Organization, underweight in low-income countries is a major health risk in infants and young children.

Sub-Saharan Africa has one of the most serious rates of chronic malnutrition in the world. In Zambia, chronic malnutrition or stunting affects 45 % of the under five children. This remains the most common nutritional disorder, being slightly above the Sub-Saharan Africa's average of 42 % (CSO, 2009) and the eighth highest rate in the world (UNICEF, 2013). In addition, micronutrient deficiencies are having an enormous impact on children's health. Around 50-55% of the children in Zambia suffer from vitamin A deficiency and iron deficiency (CSO, 2009). On the contrary, indicators of acute malnutrition remain comparatively low. Five per cent of Zambian children are wasted while 15 % are underweight (CSO, 2009).

The Zambian nutrition profile shows that 60 % of households cannot afford 3 meals per day (FAO, 2009) which leads to inadequate nutrient intake and malnutrition. The same research shows that in the period 2000-2002, the dietary energy supply was only 1,905 kcal per capita/day (FAO, 2009). This clearly shows that households do not therefore meet the estimated necessary energy requirement of 2,056 kcal per capita/day. Carbohydrates such as cereals and starchy roots are the main source of energy which account for 80 % of the total energy intake (FAO, 2009). This suggests that the intake of other essential nutrients as well as protein and lipids is generally insufficient.

1.1 Rationale for study

Spirulina is a blue-green micro algae indigenous to Africa that has the potential to meet the nutrition demand of Zambians. Spirulina contains high percentage of protein, minerals, and vitamins necessary to support children's growth. The cost of production to get the same amount of protein is much lower than that of other protein-rich foods, such as soya beans and beef, see Figure 1 below.

Figure 1: Spirulina's advantage over other protein-rich foods

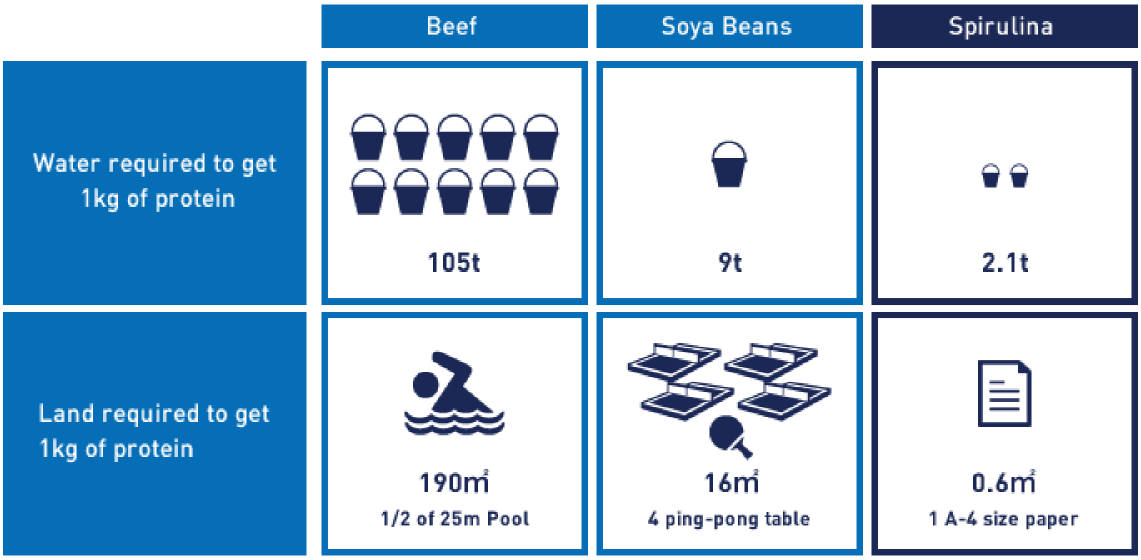

Source: Alliance Forum Foundation

Programme Against Malnutrition (PAM) and Alliance Forum Foundation (AFF) jointly conducted the first spirulina effectiveness study in Zambia from June 2012 to February 2013. The difference in growth between children who received the spirulina porridge blend (Treatment Group) and those who did not (Control Group) was measured over a for nine months.

Participating children showed that spirulina makes a statistically significant difference in height between Treatment Group and Control Group (height higher in Treatment Group). Thus, the effectiveness of Spirulina to lower the rate of stunting in Zambia was suggested. Further, there was the unexpected but positive reduction in cases of malaria among the treatment group compared to the control group during the study period.

Based on the study results, the Ministry of Health recommended the need to scale up the study to gain wider support in promoting spirulina. Therefore, this project called *Promoting Spirulina Production and Utilization in Luapula Province* was approved by CARE International in Zambia to validate the effectiveness of spirulina among malnourished children under the age of two years in Mansa and Samfya districts. The objective of the project is to increase access and consumption of spirulina to contribute to reduce the rate of stunting in children below two years of age in Luapula province by 2016.

1.2 Significance

The Zambian Government's aim is to lower the rate of stunting among children under the age of five from 45 % to 30 % by 2015. Available data shows a decline in child malnutrition from 1992 to 2007, but the slight increase again in stunting in 2010 shows that there is need for more concerted effort if Zambia to meet its 2015 target. Spirulina has the potential to expedite the Zambian Government's effort. Spirulina contains many nutrients important for child growth such as protein, beta carotene, iron, thiamine, riboflavin, Vit. B6, Vit. B12, selenium and calcium among other nutrients.

In the long-term, reducing the rate of stunting will benefit the Zambian economy. According to the National Food and Nutrition Commission (NFNC, 2012), Zambia has lost US$ 775 million in economic production due to the high rates of stunting over the 10 year period of 2004 - 2013. Further, the UNICEF report (2010) found that stunted children are more prone to diseases, often lag behind in class, become overweight in adulthood and are thus more susceptible to non-communicable diseases, and are more likely to earn less than non-stunted co-workers.[1] Although much effort has been made by government to reduce stunting, it is a daunting task to cover all the target population by public effort. This project is thus aimed at complementing government's effort in the fight against malnutrition in line with the First 1000 Most Critical Days Programme (First 1000 MCDP) priority interventions.

1.3 Aim

The second spirulina effectiveness test will be conducted as a part of the project *"Promoting Spirulina Production and Utilization in Luapula Province of Zambia"*, which is the donor funded Scaling Up Nutrition (SUN) Programme. The main purpose of the project is to scale up and validate the effectiveness of spirulina in malnourished children under the age of two years in Mansa and Samfya districts of Luapula Province.

1.4 Specific objectives

The specific objectives of the project are:

1. To verify sprulina's effectiveness on children's growth i.e. height, weight, mid-upper arm circumference
2. To verify spirulina's effectiveness on the children's immune system after analysis of the monthly morbidity data

[1] UNICEF (2010) "Improving Child Nutrition –The achievable imperative for global progress-".

1.5 Ethical Issues

This study ensures voluntary participation, informed consent, and confidentiality throughout the study. A system to avoid any risk of harm to research participants will be established.

2 Literature Review

Spirulina is a blue-green micro algae indigenous to Africa. It has been suggested that it has the ability to modulate immune function and can be used to treat several diseases (Karkos, Leong, Karkos, Sivaji and Assimakopoulos 2011). It can sustainably contribute to alleviating malnutrition because it is rich in various nutrients, is easy to produce, and can be added to many traditional foods (Hug and Weid 2011). Spirulina contains various nutrients such as protein, beta-carotene, iron, and vitamin B which are usually deficient in undernourished populations (Belay et al. 1993). Thus, the use of Spirulina is highly likely to be an effective intervention tool to tackle protein deficiency, vitamin A deficiency, and iron deficiency, which are common public health problems in Zambia.

In nutrition literature, several in vitro and animal testing studies have provided the evidence of efficacy of Spirulina on the prevention and treatment of lifestyle related diseases (Kapoor and Mehta 1993; Iwata et al. 1990; Rodriguez-Hernandez et al. 2001; Ble-Castillo et al. 2002; Suetsuna amd Chen 2001), antivirus effect (Ayehunie et al. 1998; Hernandez-Corona et al. 2002), and antioxidative effect (Upasani et al. 2001; Upasani and Balaraman 2002). A few clinical studies also revealed Spirulina’s significant efficacy on lifestyle related disease patients (Samuel et al. 2002; Parikh and Mani 2001; Ramamoorthy et al. 1996). In addition, Mani et al. (2000) proved that daily intake of five gram of Spirulina for one month improved blood haemoglobin level among anaemic adult females.

Studies on effectiveness of Spirulina on malnutrition were conducted in some countries. As little as 10g a day of Spirulina led to rapid recovery from malnutrition in children and adults in Mexico. Further, children were given 10 to 15 g/day of spirulina as a dietary supplement mixed with millet, water and spices, and they recovered in several weeks in Togo (Henrikson 1989). Good results have been obtained by treating children suffering from serious malnutrition diseases with Spirulina powder at Thuanhai Hospital, Viet Nam (Kim 1990).

The study on the use of Spirulina to HIV-infected and negative undernourished children in Burkina Faso concluded that Spirulina is a good food supplement for undernourished children. In particular, Spirulina corrected anaemia and weight loss in

HIV infected children, and even more quickly in HIV-negative undernourished children (Jacques Simpore et al. 2005). Halidou et al (2008) identified 31 references on study of Spirulina on malnutrition and reviewed 7 studies; 3 randomized controlled and 4 non-controlled trials. Spirulina had a positive impact on weight in all studies. In non-controlled trials, the other parameters: arm circumference, height, albumin, prealbumin, protein and hemoglobin improved after Spirulina supplementation.

3 Methodology

3.1 Project Sites

The project will be conducted in Kalaba camp of Mansa district and Njipi camp in Samfya district, which are among the selected 14 Phase I districts of the First 1000 MCDP. Figure 3 below shows the location of Mansa and Samfya districts.

1. Mansa
2. Samfya

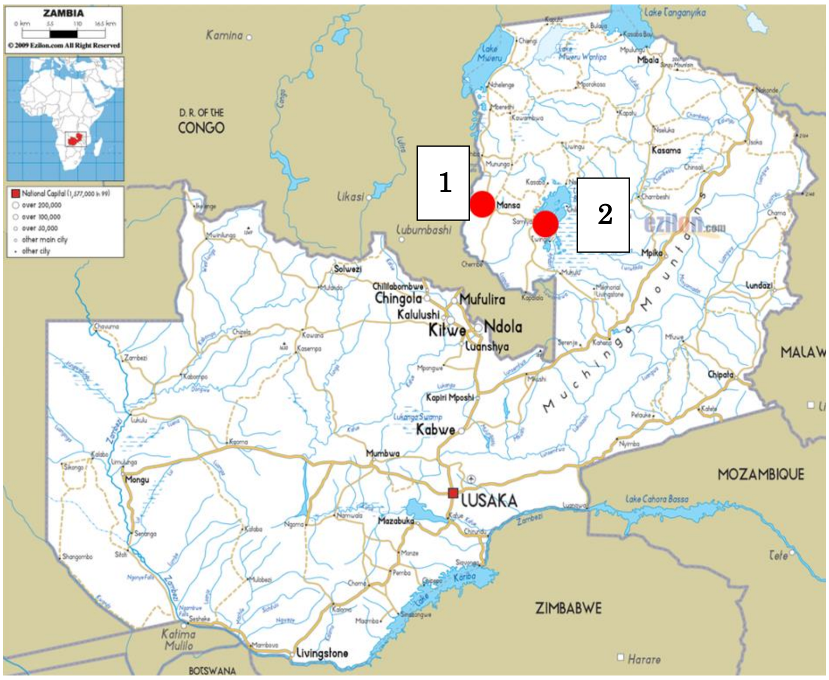

Figure 2: Map of the project sites

3.2 Target Population

The target population of the proposed study is households with children between 6 and 24 months old.

3.3 Research Process

1. Information Session

- Hold information sessions at the research area in Mansa and Samfya before the commencement of the research to inform residents about the overview and objective of the research, and benefits and responsibilities of the participants.
- Arrangement of consultation with PAM staff for households who are interested in participating, and who has a child aged under 23 months.
- Reception of signature on the consent form from households who decided to participate in the research. (See 3.7 for the detailed ethical issue).

2. Selection of Participants

- Collection of baseline data (height, weight, MUAC, socio-economic survey, dietary diversity survey, morbidity survey) from the households who signed to the consent form.
- Random Selection of a total of 500 children between the age of 6-24 months from the households who signed to the consent form.
- Random division of the children into two groups by assigning them to either treatment group or control group.

3. Intervention

- Provision of 10g of spirulina per day to the treatment group children for one year. (Spirulina will be mixed with mealie meal for porridge. The control group children will receive plain porridge blend for the same period).

4. Data Collection

- Collection of socio-economic data of participating children and their households at baseline.
- Anthropometry i.e. weight, height, and mid upper arm circumference at baseline, midline, and endline.
- Collection of morbidity history of treatment and control group children every month for one year.
- Collection of 7 day dietary recall data of treatment and control group children every three months for one year.

5. Data Analysis

- Analysis of the difference in body measurements, and morbidity history between the treatment group and the control group to see if spirulina had any effect on improving nutrition. Socio-economic data and dietary habit data will be used to exclude non-spirulina effect.

3.4 Duration

The project period is 15 months from April 2015 – June 2016

3.5 Assessment Criteria

The area of questions to be asked at the Effectiveness Test is listed in the Table below (see the draft questionnaires for details).

Area	Questions
Growth indicator	Collect the child’s height, weight and mid upper arm circumference
SES (socio-economic survey)	Ask main wage earner the consumption and expenditure on major items in past 12 months, household assets and physical access to services etc.
Dietary habits of children	Conduct 7 day dietary recall once a quarter
Morbidity History	Ask history of the child’s vaccinations and the child’s and mother’s morbidity history in the past 4 weeks (e.g. pneumonia, diarrhoea, measles, malaria)

3.6 Training

Project officers, stakeholders and child growth promoters will be sensitized on the First 1000 MCDP to enhance their understanding of nutrition issues. Further, the officers and child growth promoters will be trained in nutrition, anthropometry i.e. height, weight and mid-upper arm circumference, spirulina porridge blend preparation and cooking of other spirulina based foods and data collection. Training will promote ownership of the project and enhance accurate data collection.

3.7 Detailed Ethical issues

1. **Voluntary participation**:
   Households will make an informed choice after project sensitization and request to participate in the study. Participation in the project will be voluntary and participants’ views will be fully respected throughout the project.
2. **Informed consent**:
   Households will be availed with details on the benefits and some of the risks that could be associated with consuming spirulina using the Bemba

information sheets before they endorse their participation. Bemba consent forms will be signed as proof of having discussed the details of the project and consent to participate.

In case the participant does not speak either English or Bemba, PAM will make sure there is an interpreter to help participants understand the objectives of the study before getting the consent and proceeding with the interview.

3. **Confidentiality**:

All the documents and records concerning personal information will be strictly stored at PAM office in Lusaka or Mansa to maintain confidentiality.

4. **Anonymity**:

It will not be possible to keep participants' anonymity among AFF and PAM staff because of the need to understand the difference in lifestyle and nutritional knowledge by income and area of residence. However, personal information will be strictly confidential.

4 Budget

The research is funded by CARE International in Zambia through the donor Scaling Up Nutrition Fund. Below is the estimated budget of K1, 215, 894 as at 28th February 2015.

S/N	ITEM	AMOUNT (K)
1	Mobilization and child registration	37 920
2	Training	308 124
3	Data collection	262 380
4	Measuring equipment	71 670
5	Procurement and distribution of food items	326,406
6	Demonstrations on spirulina utilization	209,304
**7**	**Total**	**1,215,894**

5 Time Frame

The following Table shows the estimated time frame of the study.

#	Activity	Year	2015												2016					
		Month	1	2	3	4	5	6	7	8	9	10	11	12	1	2	3	4	5	6
1	Preparation																			
2	Baseline Data Collection																			
3	Start Intervention																			
4	Morbidity Data Collection																			
5	Dietary Diversity Data Collection																			
6	Baseline Data Analysis																			
7	Baseline Data Presentation																			
8	Midline Data Collection																			
9	Midline Data Analysis																			
10	Midline Data Presentation																			
11	Endline Data Collection																			
12	Endline Data Analysis																			
13	Endline Data Presentation																			

Figure 3: Project Time Frame

References

Ayehunie S, Belay A, Baba TW and Ruprecht RM (1998) ‘Inhibition of HIV-1 replication by an aqueous extract of Spirulina platensis (Arthrospira platensis)’, *Journal of Acquired Immune Deficiency Syndromes and Human Retrovirology* 18:7-12

Ble-Castillo JL, Rodriguez-Hernandez A, Miranda-Zamora R, Juarez-Oropeza MA, and Diaz-Zagoya JC (2002) ‘Arthrospira maxima prevents the acute fatty liver induced by the administration of simvastatin, ethanol and a hypercholesterolemic diet to mice’, *Life Science* 70:2665-73

Central Statistical Office, Republic of Zambia (2009) *Zambia Demographic and Health Survey*, Lusaka:Zambia Central Statistical Office.

Halidou Doudou M, Degbey H, Daouda H, Leveque A, Donnen P, Hennart P, Dramaix-Wilmet M (2008) ‘The effect of spiruline during nutritional rehabilitation’ *Systematic review, Rev Epidemiol Sante Publique 2008* 56(6):425-31.

Henrikson, R (1989) ‘Earth food Spirulina’. San Rafael, California, USA, Ronorc Enterprises, Inc.

Hernandez-Corona A, Nieves I, Meckes M, Chamorro G and Barron BL (2002) ‘Antiviral activity of Spirulina maxima against herpes simplex virus type 2’, *Antiviral Research* 56:279-85.

Hug, C. and Weid, D. (2011) *Spirulina in the fight against malnutrition; Assessment and prospects.* FONDATION ANTENNA TECHNOLOGIES, http://www.antennaindia.org/Spirulina_spirulina%20assesment%20and%20procespects.PDF (accessed 22 January 2013)

Iwata K, Inayama T and Kato T (1990) ‘Effects of Spirulina platensis on plasma lipoprotein lipase activity in fructose-induced hyperlipidemic rats’, *Journal of Nutritional Science and Vitaminology* 36:165-71

Karkos, P. D.; Leong, S. C. ; Karkos, C. D. ; Sivaji, N and Assimakopoulos, D. A. (2011) 'Spirulina in Clinical Practice: Evidence-Based Human Applications' *Evidence-Based Complementary and Alternative Medicine* eCAM:1–4

Kim,D.D.(1990) ‘Outdoor Mass Culture of Spirulina Platensis in Vietnam. *J. Appl. Phycol*. 2:179-181.

Kapoor R, Mehta U (1998) ‘Supplementary effect of spirulina on hematological status of rats during pregnancy and lactation’, *Plant Foods for Human Nutrition*

52:315-24

Mani U, A Sadliwala, U Iyer, P Parikh (2000) ‘The effect of Spirulina supplementation on blood haemoglobin levels of anaemic adult girls’, *Journal of food science and technology* 37: 642-4

National Food and Nutrition Commission (2012) *The First 1000 Most Critical Days Three Year Programme 2013-2015,* Lusaka: National Food and Nutrition Commission.

Parikh P, Mani U and Iyer U (2001) ‘Role of Spirulina in the Control of Glycemia and Lipidemia in Type 2 Diabetes Mellitus’, *Journal of Medical Food* 4:193-9

Ramamoorthy, A., Premakumari, S (1996) ‘Effect of supplementation of Spirulina on hypercholesterolemic patients’, *Journal of Food Science and Technology* 33 (2): 124–8

Rodriguez-Hernandez A, Ble-Castillo JL, Juarez-Oropeza MA, Diaz-Zagoya JC (2001) ‘Spirulina Maxima prevents fatty liver formation in CD-1 male and female mice with experimental diabetes’, *Life Science* 69:1029-37

Simpore, J.; Zongo, F.; Kabore, F.; Dansou, D.; Bere, A.; Nikiema, B. J.; Pignatelli, S.; Biondi, M. D.; Ruberto, G. and Musumeci, S. (2005) 'Nutrition Rehabilitation of HIV-Infected and HIV-Negative Undernourished Children Utilizing Spirulina' *Annals of Nutrition & Metabolism* 49: 373-80

Suetsuna K, Chen JR (2001) ‘Identification of antihypertensive peptides from peptic digest of two microalgae, Chlorella vulgaris and Spirulina plantensis’, *Marine Biotechnology* 3:305-9

United Nations Children’s Fund (UNICEF) (2013) *Improving Child Nutrition: The achievable imperative for global progress.* New York: USA. Division of Communication, UNICEF.

Upasani CD, Khera A and Balaraman R (2001) ‘Effect of lead with vitamin E, C, or Spirulina on malondialdehyde, conjugated dienes and hydroperoxides in rats’, *Indian Journal of Experimental Biology* 39:70-4

Appendix 1: Information Sheet (English)

Promoting Spirulina Production and Utilization in Luapula Province of Zambia

Prepared by:

Programme Against Malnutrition
and
Alliance Forum Foundation

28th October 2014

Copyright@ 2014 PAM All Rights Reserved

INFORMATION SHEET 1 (ENGLISH)

INFORMATION SHEET

Promoting Spirulina Production and Utilization in Luapula Province of Zambia

PSPU – Project

Prepared by:

Programme Against Malnutrition
and
Alliance Forum Foundation

Copyright@ 2014 PAM All Rights Reserved

INFORMATION SHEET 2
(ENGLISH)

RESEARCH ON SPIRULINA

Introduction - Investigators

Programme Against Malnutrition (PAM)

- Non-profit, non partisan, autonomous NGO that provides links between rural NGOs, the government and donor community
- Largest Zambian NGO with a wide network of NGOs across the country
- Formally registered on 11th November 1993

Alliance Forum Foundation (AFF)

- Nurturing new technologies and industries capable of generating a new core industry and facilitating debate on new industries for post-computer era
- Fostering debate, exchange and collaboration in various fields related to poverty alleviation in developing countries
- Founded in 1985 in California

Copyright@ 2014 PAM All
Rights Reserved

INFORMATION SHEET 3 (ENGLISH)

What is the PSPU Project

Will be in implemented in Mansa and Samfya districts

Has two Phases :

Phase I:

Check the effectiveness of spirulina on malnourished children below the age of two years

Expand spirulina recipes

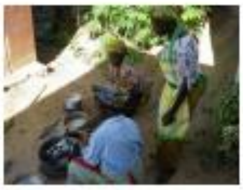

Feed spirulina

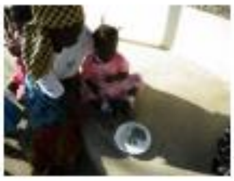

Body measurement

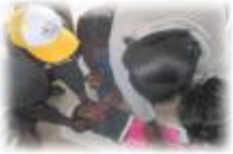

Porridge with Spirulina

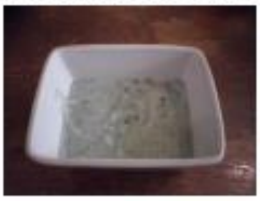

Pumpkin leaves with spirulina

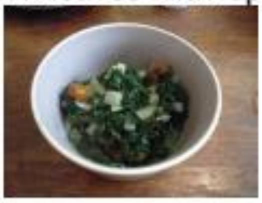

Copyright@ 2014 PAM All Rights Reserved

INFORMATION SHEET 4 (ENGLISH)

Who will be involved?

Women groups

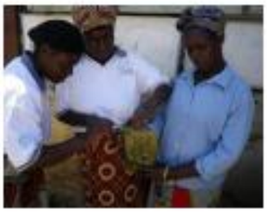

Child Growth Promoters

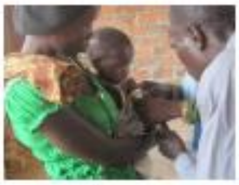

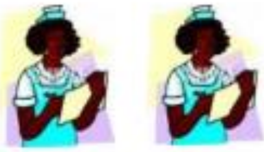

Community Nutrition Promoters

Clinic nurse

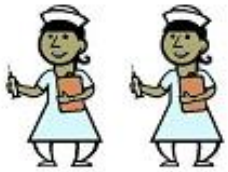

Food and Nutrition Officer

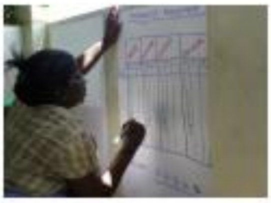

District Nutrition Committee

INFORMATION SHEET 4 (ENGLISH)

What is going to be done?

- Spirulina provision
  - Spirulina will be distributed once a month to 250 children
  - Children will eat spirulina every day for 12 months
  - Another 250 children will be the control group
  - They will eat porridge without spirulina for 12 months

- Body Measurements
  - Weight, height and MUAC
  - Will be taken three times in the year i.e. baseline, mid line and end line

- 24 Hour Recall
  - CGP and CNP will visit homes and complete the 24 hour recall sheet
  - This will be done once a quarter

- Disease record
  - Households will record disease incidence every month

- Data on living conditions will be collected once a year

Copyright@ 2014 PAM All Rights Reserved

INFORMATION SHEET 5 (ENGLISH)

What are the benefits?

- Spirulina is rich in protein Vitamins and minerals

Spirulina is a spiral micro-algae

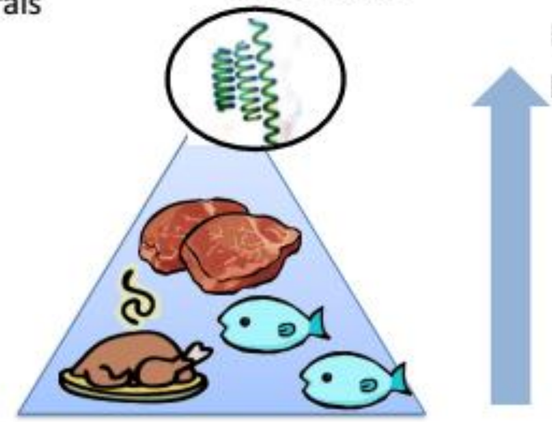

Has more protein than

- Spirulina is effective in reducing malnutrition

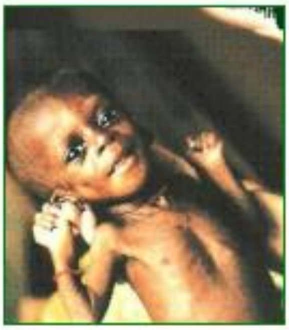

90 days of eating spirulina

Copyright@ 2014 PAM All Rights Reserved

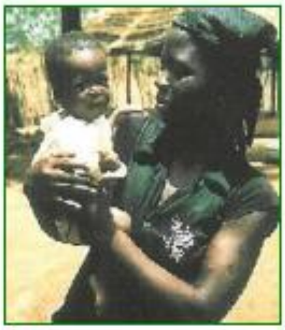

INFORMATION SHEET 6 (ENGLISH)

What are the risks?

- Spirulina is widely eaten as a food supplement across the world
- However, some people develop mild allergic reactions to spirulina as is the case with other foods
- Mild diarrhoea is the major adverse event

How can we avoid the risks?

- Check the children's stool everyday
- The stool may be more green than usual but that is not a problem
- Check the body temperature everyday

↓ If the baby has any problems because of spirulina

Stop feeding spirulina and talk to the community health worker

INFORMATION SHEET 7 (ENGLISH)

How will the participants be protected?

- **Confidentiality:**
  - All personal documents and records will be strictly stored at the RHC and PAM office to maintain confidentiality

- **Voluntary participation:**
  - Households will decide to participate after sensitization meetings are conducted
  - Participation in the project will be voluntary
  - Participants' views will be fully respected throughout the project

- **Right to withdraw**
  - All participants have the right to withdraw from the project at any time by notifying the clinic staff, CGP, CNP, PAM or AFF officers

- **Seek clarification:**
  - All participants are free to ask questions on the project design, implementation and future plans

- **Standard of care**
  - CGP & CNP will visit participants once a month to check their condition
  - If there are nay problems, participants will be referred to the clinic

INFORMATION SHEET 8 (ENGLISH)

What is the role of women group?

1. **Prepare Porridge Blend**
   Prepare spirulina porridge blend for children .

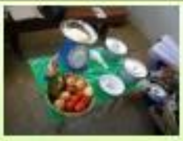
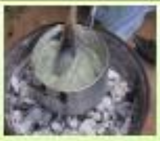

2. **Provide mealie meal mixed with spirulina to CGPs & CNPs for distribution**
   Blend spirulina, mealie meal, sugar and salt and distribute to mothers

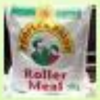

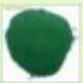
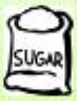

3. Record keeping
Record quantity of porridge ingredients used per month

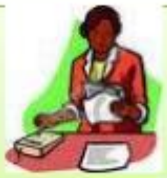

4. Monitoring
Regularly check if mothers feed their children properly

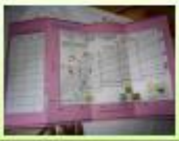

Copyright@ 2014 PAM All Rights Reserved

INFORMATION SHEET 9 (ENGLISH)

What is the role of family members?

1. Participant in training
Learn how to prepare spirulina recipes for children

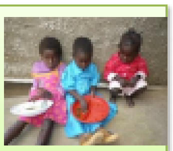

2. Cook and feed the child regularly
Cook food with spirulina for children

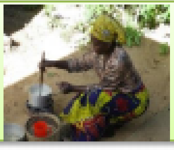

3. Check children's health
Pay attention to children's health every day

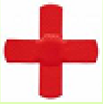

4. Attend under five clinics
Attend under five clinics every month for body measurements

Copyright@ 2014 PAM All
Rights Reserved

INFORMATION SHEET 10 (ENGLISH)

PHASE II

❑ **Participate in spirulina production**

- Identify women group representatives to participate in spirulina production
- Identify site for spirulina production
- Seek permission to use land for spirulina production
- Small scale spirulina production will be promoted

❑ **Training in spirulina production**

- Demonstration on how to make spirulina ponds
- Stock ponds with spirulina
- Manage ponds
- Harvest, dry and sell spirulina in the community and at the markets

❑ **Who will conduct the training**

- Alliance Forum Foundation and University of Zambia

Copyright@ 2014 PAM All Rights Reserved

INFORMATION SHEET 11 (ENGLISH)

PROGRAMME AGAINST MALNUTRTION

Principal Investigator: Maureen Chitundu
178 Parirenyatwa Road, Fairview
P. O. Box 30599, Lusaka, Zambia.
Tel: + 260 211 235941/2
Fax No. +260 211 235939

ALLIANCE FORUM FOUNDATION

Principle Investigator: Yumi Ota
Yumi Ota or Ms. Nakauchi???
Nihonbashi-Hongokucho 4-4-20, Mitsui 2nd Annex 7F, Chuo-ku, Tokyo
Tel: +81-3-6225-2795
E-mail: ?????

UNIVERSITY OF ZAMBIA
BIOMEDICAL RESEARCH ETHICS COMMITTEE

Ridgeway Campus,
P. O. Box 50110, Lusaka, Zambia.
Tel: +260 211 256067
Fax No. +260 211 250 753
Email: unzarec@zamtel.zm

Contact Details

Appendix 2: Information Sheet (Bemba)

Ukutungilila ubulimi bwa Spirulina elyo nefya ku ibonfya ku citungu ca Luapula Province Mu calo ca Zambia.

Abamipekanishako Niba:

Programme Against Malnutrition naba Alliance Forum Foundation

29th December, 2014

Copyright@ 2014 PAM
All Rights Reserved

UBUTANTINKO BWALENGA CIIMO
(ICIIBEMBA)

Ukutungilila ubulime bwa Spirulina elyo nemibofese ku citungu ca Luapula Province Mu Calo Ca Zambia.

PSPU - Project

Abapekenye bamukabungwe ka:

Programme Against Malnutrition
Naba
Alliance Forum Foundation.

Copyright@ 2014 PAM
All Rights Reserved

UBUTANTINKO BWALENGA CIIBILI
(ICIBEMBA)

Ukuceceta palwa Spirulina

Bulondoloshi -Nokufwailisha.

Akabungwe Ka Programme Against Malnutrition (PAM)

❑Takapanga impiya mu mibombele yakako, takaibimba mufikansa fya calo, takakwata uko kashitula lelo kabelako ukwikatishanya kwa tubungwe utwabela mu ncende sha mishi nobuteko elyo nokubi kapofye notubungwe utuleta ubwafwilisho mubwina mupalamano.

❑Akabungwe akalamba nganshi aka bomba no tubungwe utwingi nganshi mpanga mu calo ca Zambia.

❑Akalembeshewe pa bushikubwa bwa lenga Ikumii na bumo ,mumweshi wa kasaka ntobo mu mwaka wa 1993.

Akabungwe ka Alliance Forum Foundation (AFF)

❑Ukupakamisha ifyabuyantanshi mukufuntula utwampani mufya kupangapanga elyo nokuleta inshila yakykansana pakuleta utwampani utupya ukutampa uku Bomfya ba computer ukwenda ne nshita isonde ufikilepo.

❑Ukukoselesha inshila shaku sambilisha pakupwisha insala kufyalo ficili filetumpuka mu buyantanshi.

❑Akakabungwe Katampile mumwaka wa 1985 Ku ncende ya California.

Copyright@ 2014 PAM

UBUTANTINKO BWALENGA CIITATU
(ICIBEMBA)

Imuliomo ba PSPU balefya ukubomba

Imilimo ika bombewa ku Mansa elyo nakwi boma lya Samfya Mufipande fibili:

Mukutendekelapofye

- Icakubalilapo kuceceta imibombele ya spirulina mubana abalwala ubulwele bwansala abashilakwanya myaka ibili.

ukufusha kwa spirulina mu imilila

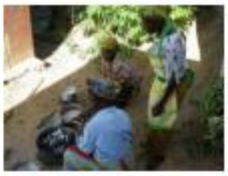

ukubalisha spirulina

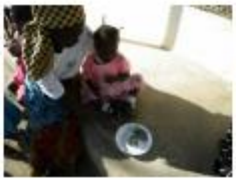

iicipimo cabutali

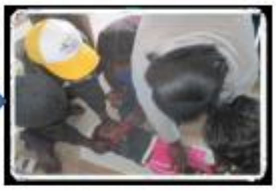

ukusankanya musunga elyo na spirulina

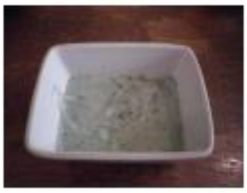

chibwabwa nokusankana na spirulina

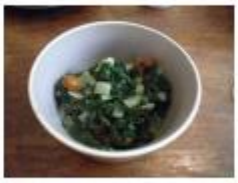

Copyright@ 2014 PAM

UBUTANTINKO BWALENGA CIINE
(ICIBEMBA)

NIBANI BALINOKUIBIMBAMO?

Utubungwe twabanamayo

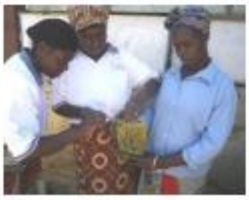

Utubungwe

Abakutungilila imikulile ya bana

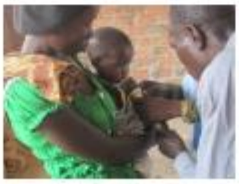

utwakutungilila twamubwina mupalamano

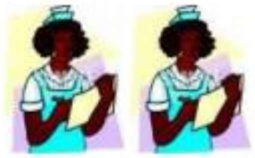

Ba Nurse Bapa Chipatala chi nono

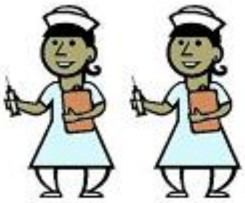

umubomfi uleyangalila ifyakulya ifya kufilisha umubili

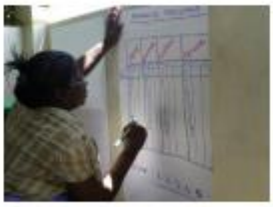

DISTRICT NUTRITION COMMITTEE

Copyright@ 2014 PAM

UBUTANTINKO BWALENGA CIINE
(ICIBEMBA)

Ifi fwile ukucitwa finshi?

☐ **Eyo spirulina ya kulapelwa**

▪Kufwile ukusalanganya spirulina cila mweshi kubana abanono ukufika nempendwa 250.

▪Abana abanono bafwile ukulya spirulina cila bushiku pa myeshi ukufika ikumi limo na yabili.

▪Ukulundapo nabana 250 ebafwile ukutungilila mu milile isuma elyo nokucecetwa.

▪Abana bafwile ukulya umusunga uwabula spirulina pa mweshi ikumi limo nayabili.

☐ **Ukupimwa kwa Mubili**

•Ukufina, ubutali elyo na muac

•Kufwile ukusendwa imiku itatu pa mwaka ukutampila panshi,m pakati elyo nakumpela.

☐ **UBUSHIKU BONSE**

•Utubungwe ututungilila imikulile yabana abanono utwitwa child growth promoters mukwipifya (CGP) Elyo Nokubika pofye notubungwe twabu mu bwina mupalamano utukoselesha pa milile isuma itwitwa community nutrition promoters mukwipifya (CNP) tufwile ukutandalila amayanda nokipwisha umulimo wabushiku bonse ukulingana nefili ubutantiko.

•Ifi fifwile ukucitwa cilao cipandwe cabutantiko.

☐ **Ukulemba Amalwele**

•Cila Nganda ukulemba amalwele bakumanya cila mweshi

☐ **Ifishinka fyalembwa pafilengwa fifwile ukupokwa cila mwaka**

Copyright@ 2014 PAM

UBUTANTINKO BWALENGA FISANO (ICIBEMBA)

Spirulina yaba nga limbwe limbwe iya nyongana

☐ **Bushe bunonshi nshi bulimo?**

•Ifintu mwinganonkelamo muli spirulina yalikwata sana ifilyo ifisuma mukati apali ama ubusanshi(protein) ukukusha bwino umubili, ama vitamins uku mici-ngilila kumalwele na minerals ukukosha umubili.

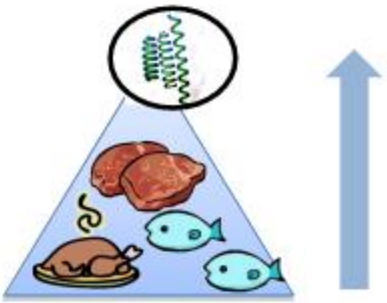

Yalikwata ubusanshi wamubili ubwingi

☐ **Spirulina ila pwisha nokucefya ko ubulwele bwa nsala.**

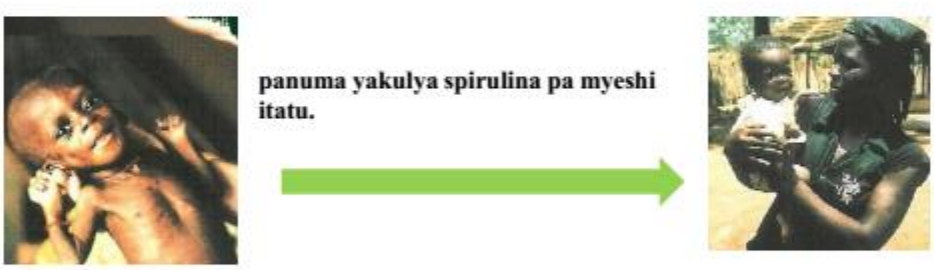

Copyright@ 2014 PAM

UBUTANTINKO BWALENGA MUTANDA
(ICIBEMBA)

BUSHE BUSANGO NSHI BULIMO?

- Spirulina ilalilwa isonde lyonse inge cakulya capambalifya pa fiakulya fimbi ifyo umuntu alile.
- Lelo abntu bambi ilatampa ukubakana nokubaletela ifyakubafuluma pa mubili inge fyo nefilyo fimbi ficita.
- Ubwafya bwakupolomya ebwa seka ukumonekela mu bantu aboikana.

TACITA SHANI PAKUCICINTILA UBU BWAFYA?

- Ukulaceceta imisu cila bushiku.
- Imisu nga ilamoneka iya katapa katapa ninshi pali ubulema, lelo ubutebwafya sana.
- Ceceteni umubili wa mwana umwana ingatakwete ubwafya ubuli bonse.

Nga cakutila umwana akawat ubwaya elyo alebomfya spirulina.

Lekeni ukulisha umwa spirulina elyo landeni nabo bomfi aba mu bwina mu palabano

Copyright@ 2014 PAM

UBUTANTINKO BWALENGA CIINE LUBALI
(ICIBEMBA)
BUSHE ABALI NOKUSENDAMO ULUBALI BALINOKUCINGILILWA MU MUSANGO NSHI?

IFYANKAMA
•Ifipepala fyonse nefyalembwa fifwile ukusungwa bwino munkama pama office yaba fipatala finono ifya mu mushi elyo nama office yambi ayaba PAM.

Abalefya ukusendamo ulubali bafwile ukuipeleshyafye.
•Aba mu mayanda kuti batumpamofye nga cakutila basambilishiwa.
•Ukusendamo ulubalimuli uyumulimo kufwile kwabafye mukulipela.
•Imilandile namatontonkanyo yabo abale sendamo ulubali ifwile ukucindikwa mpakafye nokumpela yamulimo onse.

Insambu shakufumamo
•Abalesendamo ulubali muli uyu milimo nabakwata insambu shakuleka atemwa ukukanakankanypo no mulimo inshita fye iliyonse cikulifye ba ishibisha abamiciputulwa ca bumi elyo naba CGP, CNP,PAM na ngula ba bonfye ba ukabungwe ka AFF.

Ukufwaya Ukwishiba ifishinka
•Abo balesendamo ulubali abantungwa ukwipusha ifipusho pa mulimo ifyo uli, ifya kucita pakuti ufukilishiwe nama pange yakuntanshi.

Kufyo ukusungwa kwafikapo kufwile ukuba
•Ukubungwe ka CGP naka CNP bakalapitamo kutandalila abalesendamo ulubali cila mweshi umuku umo no mukuceceta ubumi bwa bantu.
•Inga kuli ubwafya ubuli bonses, abalebomba uyu mulimo kuti ba batuma ku cipatala.

Copyright@ 2014 PAM

UBUTANTINKO BWALENGA CIINE KONSE KOSE (ICIBEMBA)

Bushe Lubali Nshi Banamayo Bakwete Mutubungwe?

1. Pekanyeni umusunga uwa spirulina uwabana banono munshilayabusaka.

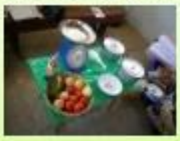

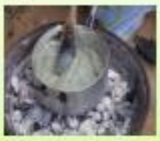

2. Pekanyeni ubunga elyo musankanye na spirulina kaliba CGPS elyo nakuli ba CNPS pakuti basalanganye.

Sankanyeni spirulina, ubunga, sugar elyo na mucele eclyo nokusalanganya kali banamoyo.

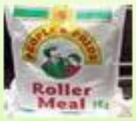

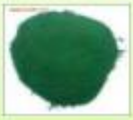

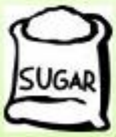

3. Ukusunga ifishinka fyamibombele

Lebeni icipimo chabukumu icifwile ukuba mu musunga mulebomfewa cila mweshi

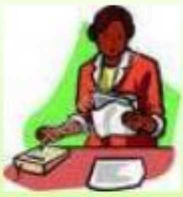

4. Ukusopa

Cila nshita mufwile ukulececeta kufyo banamayo balelisha abana babo mufyo cifwile ukuba.

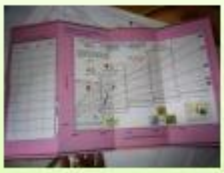

Copyright@ 2014 PAM

UBUTANTINKO BWALENGA PABULA
(ICIBEMBA)
Bushe mulimo nshi abamu yanda bakwata ?

1.Sendenimo ulubali mukusambilishiwa sanbilileni ifyakupekanya spirulina iya bana banono

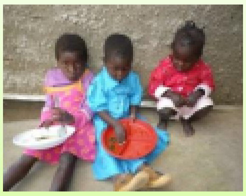

2. Ipikeni elyo nokulisha abana banono cila nshita.
Elyo mwaipika icakulya na spirulina c bana banono

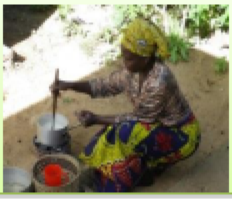

3. Ceceteni ubumi bwabnana abanono, poseniko amano sana kubana abanono ukumona ifili ubumi bwabo cila bushiku

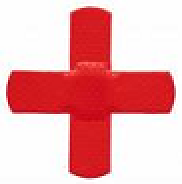

Bafwile ukulaya ku under five ku cipatala.
Ukuya ku cipimo ku under five ku cipatala bacecete umubili.

Copyright@ 2014 PAM

UBUTANTINKO BWALENGA EKUMI LIIMO (ICIBEMBA)

ULUTAMPULO LWA CIBILI

Ukusendamo ulubali mumulimo wakupanga spirulina
Fwailikisheni utubungwe utwaba banamayo utwinga sendamo ulubali mukupekanya kwa spirulina.
Fwayeni incende apo mwinga pekanisisha spirulina.
Pokeni insambu kubakwete impanga apo mwinga limina spirulina.
Ubulimi bunono ubwa spirulina kuti bwatungililwa.

Amasambililo palwa bulimi bwa spirulina
Ukubalondolwela ifya kupekanya ifishiba ifya kusungilamo spirulina.
mufwile ukulacecetwa Ifshiba umuli spirulina .
Ifishoba fifwile ukulasungwa bwino.
Somboleni, yanikeni elyo shitiseni kubena mupalamano elyo naku malikete

Abafwile ukusambilisha aya amasambililo
Alliance Forum Foundation and University of Zambia

Copyright@ 2014 PAM

UBUTANTINKO BWALENGA EKUMI NA LIIMO (ICIBEMBA)

PROGRAMME AGAINST MALNUTRTION
Principal Investigator: Maureen Chitundu
178 Parirenyatwa Road, Fairview
P. O. Box 30599, Lusaka, Zambia.
Tel: + 260 211 235941/2
Fax No. +260 211 235939

ALLIANCE FORUM FOUNDATION
Principle Investigator: Yumi Ota
Nihonbashi-Hongokucho 4-4-20,
Mitsui 2nd Annex 7F, Chuo-ku, Tokyo
Tel: +81-3-6225-2795
E-mail: y-ota@allianceforum.org

UNIVERSITY OF ZAMBIA
BIOMEDICAL RESEARCH ETHICS COMMITTEE

Ridgeway Campus,
P. O. Box 50110, Lusaka, Zambia.
Tel: +260 211 256067
Fax No. +260 211 250 753
Email: unzarec@zamtel.zm

Copyright@ 2014 PAM

Appendix 3: Consent Form (English)

Participant Identification Number: …………………………

CONSENT FORM

Title of Project: Promoting Spirulina Production and Utilization in Luapula Province of Zambia

Please Tick to Confirm

Name of Project Officer: ……….…………………………

1. I confirm that I have read and understood the information sheet dated.................................. for the above project. ☐

2. I had the time to consider the information and to ask questions which were answered satisfactorily. ☐

3. I understand that my participation is voluntary and that I am free to withdraw at any time, without giving any reason, without my medical care or legal rights being affected. ☐

4. I understand that relevant sections of any of my data collected during the project may be looked at by responsible individuals from Programme Against Malnutrition (PAM), Alliance Forum Foundation (AFF) or from regulatory authorities, where my data may be relevant during the course of this research. I give permission for these individuals to have access to my records. ☐

5. I agree to take part in the above research project.

**Name of Participant**	**Date**	**For/Thumb Print**
**Name of Caregiver**	**Date**	**Signature**
**Witness**	**Date**	**Signature**
**Project Officer**	**Date**	**Signature**

When complete: **1** copy for participant: **1** copy for Rural Health Centre File: **1** (original) copy for PAM

Contacts

Programme Against Malnutrition (PAM)

- Principal Investigator: Mrs. Maureen Chitundu
- Contact Address: 178 Parirenyatwa Road, Fairview
  P.O. Box 30599 Lusaka, Zambia
- Telephone No: +260 211 235941/2
- Fax No: +260 211 235939
- Email: cm.maureen@yahoo.com

Alliance Forum Foundation

- Principal Investigator: Ms. Yumiko Ota
- Contact Address: Nihonbashi-Hongokucho 4-4-20, Mitsui 2nd Annex
  7F, Chuo-ku, Tokyo
- Telephone No: +81-3-6225-2795
- Email: spirulina@allianceforum.org

The University of Zambia
Biomedical Research Ethics Committee

- Contact Address: Ridgeway Campus, P.O. Box 50110, Lusaka, Zambia
- Telephone No: +260 211 256067
- Fax No: +260 1 250753
- Email: unzarec@zamtel.zm

Appendix 4: Consent Form (Bemba)

29th December, 2014

Inamba ya kwishibilako abale itumpama: _____________________

ICIPEPALA CAFISHINKA ICAKUBALI LAPO

Umutwewa Mulimo: Ukutungilia ubulimi bwa spirulina enimiboshe citunguca Luapula

Ishina lya mubomfi Uleyangilila uyo mulimo: ....

Chongeni Mukabokoshi Ukusuminesha

1. Nasuminisha ukutila nalibelenga nokumfwikisha ifishinka filipali icicipepala ica kufwatika pokabil nefe lembelwepo................................. Pali uyu Mulimo ☐
2. Nalikwata inshita iya kupitulukamo muli ififishinka elyo nokwipusha amepusho nokwasuka nail yasukwa fye bwino ☐
3. Ninjishiba ukutila ukubomba uyumulimo wakuipela elyo nokutila ninkwata insambu inshita iliyonse ukuleka ukwabula ukungipusha ico nacitila ifyo ukwabula ukupufyanya insambu sha bumi elyone mikaule. ☐
4. Elyo nsumina nokumfwikisha kufishinka fyonse ifipokelwe muliuyu mu limo kuti bafilolekeshapo ku bantu ukufuma mu tubungwe pali PAM ,AFF Elyo nakuba le twangalila abe abenga sendamo i fili nobukumu ifya sangwilwe Mukuceceta Kwandi. Nimpela insambu kului abo Bantu numbwile uku kwata shuko kufyo nabombele uyu umulimo. ☐
5. Ninshumina ukusendamo ulubali muli uyu mulimo wakuceceta.

Nga Mwapwisha Ukulemba: Ichikope Chimo Ichenu Mwe Ba Itumpilemo: Ichikope Chimo Chaba Health Centre: Elyo limbi mupele ba PAM.

1 | Page

____________________	___________	___________________
ISHINA LYA LULESENDAMO	UBUSHIKI	APA KUFWATIKA ICIKUMO
____________________	___________	___________________
ISHINA LYA KWA KANALILA	UBUSHIKI	APA KUSAINA
____________________	___________	___________________
ISHINA LYA BAKANGALILA WA MULIMO	UBUSHIKI	APA KUSAINA

CONTACTS

PROGRAMME AGAINST MALNUTRTION
Principal Investigator: Maureen Chitundu
178 Parirenyatwa Road, Fairview
P. O. Box 30599, Lusaka, Zambia.
Tel: + 260 211 235941/2
Fax No. +260 211 235939

ALLIANCE FORUM FOUNDATION
Principle Investigator: Yumi Ota
Nihonbashi-Hongokucho 4-4-20, Mitsui
2nd Annex 7F, Chuo-ku, Tokyo
Tel: +81-3-6225-2795
E-mail:y-ota@allianceforum.org

UNIVERSITY OF ZAMBIA
BIOMEDICAL RESEARCH ETHICS COMMITTEE
Ridgeway Campus, P. O. Box 50110, Lusaka, Zambia.
Tel: +260 211 256067Fax No. +260 211 250 753
Email: unzarec@zamtel.zm

Appendix 5: Interview Guide

Guide for Interviewers

This document was created for interviewers to review before going into the field for an interview.

1. **Courtesy**
   - Dress up neatly on the interview days.
   - Be polite to households you visit. Pay particular attention and respect their schedule and health. For example, ask convenient time for conducting the interview. If the interviewee expresses any health issue, schedule for another day of visit and do not push them.
   - Be clear on the issues of confidentiality and rights of the interviewee before questioning by using the information sheet.
2. **Plan**
   - Note the time when to collect particular data throughout the year. Especially, be aware of three different types of questionnaires—monthly, quarterly, and semi-annually, so that you can get the right information at the right time.
   - Schedule when to visit which zone every month in consultation with the Rural Health Centre.
3. **Review**
   - Review the questionnaire sheet each day before and after going into the field.
   - Summarize what each questionnaire is all about so that you do not get confused in front of the interviewee.
   - Take note of any challenges encountered in the field and share them with colleagues and PAM staff for appropriate decisions to be made.

Appendix 6: Body Measurement Record Format

HHID	Year	Month	Name of Child	Age of Child (in months)	Sex of Child	Measurer ID	Day of Measurment	Officer ID of Data Entry	Height (cm)	Weight (kg)	Mid Upper Arm Circonference (cm)
1											
2											
3											
4											
5											
6											
7											
8											
9											
10											
11											
12											
13											
14											
15											
16											
17											
18											
19											
20											
21											
22											
23											
24											
25											
26											
27											
28											
29											
30											
31											
32											
33											
34											
35											
36											
37											
38											
39											
40											
41											
42											
43											
44											
45											
46											
47											
48											
49											
50											

Appendix 7: Socio-Economic Status Questionnaire

Draft Ver.4.1

Spirulina Baseline Survey in Zambia
Household-Level Survey (April 2015)

Alliance Forum Foundation, Japan (AFF) and Programme Against Malnutrition, Zambia (PAM)

Date of interview Date: ……… Month: …………. Year: _________

Interviewed by: _____________________ Interviewer ID _____________

Date checked: Date: …….. Month: ………… Year: _________

Checked by: __________________ Officer ID ______

Date entered: Date: ………… Month: ……….. Year: _________

Entered by: __________________ Officer ID ______

District (current definition):_42=Mansa, 45=Samfya

Code: _____________

Camp: ____________________ Code: _____________

Village: ____________________ Code: _____________

Household ID: ____________________

Household Head Name in 2015: ___________________________

Main Respondent Name: _________________________________ HH member ID of main respondent: ______________

Ethnicity of HH head: __________________ Code: __________

Religion of HH head: ___________________ Code: ___________

Phone Contact: ____________________________ Owned by Household Yes □ / No □

Instruction for interviewer

Please ask questions to the mother.

General codes

-9=Not applicable	-7 = Refuse to answer	-5 = Missing Data
-8 = Don't know	-6 = Don't recall	1= Yes, 0= No

Section 1. Demography

A "household" includes all members who eat from the same pot and share income and other resources. Also include workers or servants as members of the household if they stayed in this household at least one month in the last 12 months. Use an extra sheet if necessary.

Household ID	HH member ID	Name	Sex<br>1=M<br>2=F	Months lived in the home in the last 12 months? **(If D2=0, then skip D6-10 and go to** D11 and then **Section 2)**	If less than 12 months (**D2<12**), why? **Code below**	Year born	**Month born**	Relation to current head **Code below**	If this household member's biological mother lives in this HH, put HH member **ID of the biological mother.**	If this household member's biological father lives in this HH, put HH member **ID of the biological father.**	If this household member is married and the spouse lives in this HH, put **ID of the spouse**.	Is this household member currently attending school? 1=Yes No=0	Highest grade completed **Code Sheet**
**ID**	**HH member ID**	**Name**	**D1**	**D2**	**D3**	**D4**	**D5**	**D6**	**D7**	**D8**	**D9**	**D10**	**D11**

Code for D3:
1=To find a job
2=To attend school
3=Married away
4=Passed away
5=Just born in
6=Just married in
7=Just joined w/o specific reason 8=Orphaned
9=Other (specify)

Code for D6:
0=Head
1=Spouse 2=Parent
3=Child
4=Grand child
5=Nephew/Niece
6=Son/daughter-in-law
7=Brother/Sister
8=Wife 2
9=Wife 3
10=Other relative
11=Other non-relative

Section 2-a. Child's characteristics

Household ID	Name of Child	Sex of child<br><br>1=M<br>2=F	Year born	Month born	Date born	Age in months	In the first 40 days after the baby was born,<br>did you or the baby receive any follow-up care (e.g.: asking about or examining baby's health)<br>1=Yes, both mother &baby<br>2=Yes, baby only<br>3=Yes, mother only<br>4=No	**Compared with other infants, was this child bigger, smaller or similar in size at birth?<br>1= much bigger<br>2= bigger<br>3= similar<br>4= smaller<br>5= much smaller<br>-8= Don't Know**	Was this child Weighed at birth?<br>1=Yes<br>0=No	**How many kilograms was […] at birth? (e.g., 3.5kg; if not known fill in -8)**
**ID**	**Name**	**C1**	**C2**	**C3**	**C4**	**C5**	**C6**	**C7**	**C8**	**C9**

Section 2-b. Child's characteristics Vaccination

		BCG (Bacillus Calmette-Guerin)		Polio						DPT (Diphtheria, Pertussis, Tetanus)						Measles	
Household ID	Has this child ever had vaccination? 1=Yes 0=No[If No, skip to **next section**]	Has this child ever had BCG vaccination? 1=Yes 0=No	At what age in months did this child receive BCG vaccination?	Has this child ever had Polio at birth vaccination? 1=Yes 0=No	At what age in months, did this child receive Polio at birth vaccination?	Has this child ever had Polio 1 vaccination? 1=Yes 0=No	At what age in months, did this child receive Polio 1 vaccination?	Has this child ever had Polio 2 vaccination? 1=Yes 0=No	At what age in months, did this child receive Polio 2 vaccination?	Has this child ever had DPT 1 vaccination? 1=Yes 0=No	At what age in months, did this child have DPT 1 vaccination?	Has this child ever had DPT 2 vaccination? 1=Yes 0=No	At what age in months, did this child have DPT 2 vaccination?	Has this child ever had DPT 3 vaccination? 1=Yes 0=No	At what age in months, did this child have DPT 3 vaccination?	Has this child ever had Measles vaccination? 1=Yes 0=No	At what age in months, did this child receive Measles vaccination?
**ID**	**V1**	**V2**	**V3**	**V4**	**V5**	**V6**	**V7**	**V8**	**V9**	**V10**	**V11**	**V12**	**V13**	**V14**	**V15**	**V16**	**V17**

Section 2-c. Child's characteristics Morbidity History

Household ID	Pneumonia				Measles & Malaria						HIV	
	Has (NAME of the child) ever had a cough at any time? [If the answer to CM1 is No, skip CM2-CM4, and go to CM5] 1=Yes 0=No	[If "Yes" to CM1,] did he/she breathe faster than usual with short, rapid breaths or have difficulty breathing? 1=Yes 0=No	[If Yes to CM2,] did you take him/her to a hospital? 1=Yes 0=No	[If Yes to CM3,] has he/she ever been diagnosed with Pneumonia and been hospitalized? 1=Yes 0=No	Has (NAME of the child) ever had a high fever? [If the answer to CM5 is No, skip CM6-CM10, and go to CM11] 1=Yes 0=No	If yes to CM5, did he/she have rash on his /her body after fever? 1=Yes 0=No	Has he/she ever been diagnosed with Measles? 1=Yes 0=No	If yes to CM5, did he/she suffer from Malaria? 1=Yes 0=No	If No to CM8, did s/he have a fever with a chill, shaking and nausea? 1=Yes 0=No	If No to CM8, did s/he have a swing between low temperature and high temperature? 1=Yes 0=No	Has he/she ever taken HIV test? 1=Yes 0=No	Was the result of HIV test positive? 1=Yes 0=No
**ID**	**CM1**	**CM2**	**CM3**	**CM4**	**CM5**	**CM6**	**CM7**	**CM8**	**CM9**	**CM10**	**CM11**	**CM12**

Section 2-d. Breast feeding practice and Caretaker

Instruction: *MAIN care taker is the person who physically takes cares of the target child usually, NOT financially. Example includes (Cooking her/his meals, stay with her/him when (s) he is sick etc.)

Household ID	Breast feeding				Caretaker					
	Have you ever breast fed this child? **1=Yes** **0=No** [If No, skipF2-F4 and go to **T1**]	Was this child breast fed last month? **1=Yes** **0=No**	How long have you **exclusively** breast fed this child? (in months)	How long have you breast fed this child? (in months)	HH member ID of MAIN caretaker* ? [use the HH member ID from section 1 &READ instruction above]	What is the child's relationship with the MAIN care taker? **See code below**	Gender of MAIN caretaker? 1=M 2=F	Age of MAIN caretaker?	What is the Highest grade completed by the MAIN care taker? **[Use the Code Sheet]**	How many children under the age of 5 does this MAIN care taker take care of, including this child?
**ID**	**F1**	**F2**	**F3**	**F4**	**T1**	**T2**	**T3**	**T4**	**T5**	**T6**

Code for T4:

1=Mother 2=Father
3=Grand mother
4=Grand father
5=Uncle
6=Aunt
7=Brother
8=Sister
9= Others (Specify)

Section 2-e. Motor Development

	Motor Development							
Household ID	Can this child stand with assistance?<br>1=Yes<br>0=N,o [If the answer to E1 is No, skip E2-E8 and go to section 3-a]	At what age in months, did she start standing with assistance?	Can this child stand without assistance?<br>1=Yes<br>0=No [If the answer to E3 is No, skip E4-E8, and go to section 3-a]	At what age in months, did s/he start standing without assistance?	Can this child walk with assistance?<br>1=Yes<br>0=No [If the answer to E5 is No, skip E6-E8, and go to section 3-a]	At what age in months, did she start walking with assistance?	Can this child walk without assistance?<br>1=Yes<br>0=No [If the answer to E7 is No, skip E8 and go to **Section 3-a]**	At what age in months, did s/he start walking without assistance?
**ID**	**E1**	**E2**	**E3**	**E4**	**E5**	**E6**	**E7**	**E8**

Section 3-a. Mother's characteristics

		Demography						Health							
Household ID	Name of Mother	HH member ID of the mother	Age of mother	Height in cm of mother	Current weight in kg of mother	Relation to household head **See Code below**	Highest grade completed **Use Code Sheet in the last page**	In general, how has your health been in the last 2 years? 1=Very healthy 2=Somewhat healthy 3=Somewhat unhealthy 4=Unhealthy [Whatever the answer to M7 is, ASK	In the previous 4 weeks, how many days were you [...] not able to perform daily chores or work due to poor health? [Enter 0 if the mother has worked all last 4 weeks. If M8=0, skip M9-12, and go to M13]	[If the answer to M8 is 1 or more,]What was the cause of M8? [SEE CODE BELOW, ANSWER ALL THAT APPLY]	Where did you [...] seek treatment? [SEE CODE BELOW, ANSWER ALL THAT APPLY] If M10=W, ask M12 and M13. If M10 is other than W, ask M11 only.	If H7 ≠ W, how much did you [...] pay in total out of pocket for consultation, treatment and medicine? [IF MORE THAN ONE ILLNESS OR VISIT, SUM ALL COSTS]	If H7=W, what factors led [...] to not seek for treatment or medicine? [SEE CODE BELOW]	Have you ever given live birth to a child who died before the age of 2? 1=Yes; 0=No	[If Yes to M12], what was the cause of the death? USE code below
**ID**	**Name**	**M1**	**M2**	**M3**	**M4**	**M5**	**M6**	**M7**	**M8**	**M9**	**M10**	**M11**	**M12**	**M13**	**M14**

Code for M5:
0=Head
1=Spouse 2=Parent
3=Child
4=Grand child
5=Nephew/Niece
6=Son/daughter-in-law
7=Brother/Sister
8=Wife 2

Code for M9:
A= fever
B= coughing
C= diarrhoea
D= injury/accident
E= chronic disease
F= others (specify)

Code for M10:
W= did not seek treatment or medicine
A= government hospital
B= government health centre (levels 3 and 4: sub county)
C= government health post (levels 1 and 2: LC1/Parish)
D= private doctor/hospital/clinic
E= pharmacy/drug shop
F= traditional practitioner
G= LC1 trained person for getting "home pack"
H= other (specify)

Code for M11:
1=Did not need treatment or medicine as illness was not serious
2= Did not want to visit health facility alone
3= Did not want to visit health facility because they are too far
4= Did not want to visit health facility because they do not provide good service or medicine
5= Did not want to visit health facility because waiting time is too long
6= Did not want to visit health facility because staff attitude is not good
7=Wanted to visit health facility, but did not have money
8=Wanted to visit health facility, but family did not allow
9= other (specify)

Code for M14:
1=Measles
2=Malaria
3= Diarrhoea
4= Pneumonia
5=Traffic Accident
6= Cholera
7= Others (Specify)

Section 3-b. Mother's morbidity history

Household ID	Pneumonia				Measles & Malaria						HIV	
	Have you ever had a cough at any time? [If the answer to MM1 is No, skip MM2-MM 4, and go to MM5] 1=Yes 0=No	[If "Yes" to MM1,] did you breathe faster than usual with short, rapid breaths or have difficulty breathing? 1=Yes 0=No	[If Yes to MM2,] did you go to hospital to seek a treatment? 1=Yes 0=No	[If Yes to MM3,] have you ever been diagnosed with Pneumonia and been hospitalized? 1=Yes 0=No	Have you ever had a high fever? [If the answer to MM5 is No, skip MM6-MM10 , and go to MM11] 1=Yes 0=No	[If yes to MM5,] did you have rash on your body after fever? 1=Yes 0=No	Have you ever been diagnosed with Measles? 1=Yes 0=No	[If yes to MM5,] did you suffer from Malaria? 1=Yes 0=No	[If No to MM8,] did you have a fever with a chill, shaking and nausea? 1=Yes 0=No	[If No to MM8,] did you have a swing between low temperatur e and high temperatur e? 1=Yes 0=No	Have you ever taken an HIV test? 1=Yes 0=No	Was the result of HIV test positive? 1=Yes 0=No
**ID**	**MM1**	**MM2**	**MM3**	**MM4**	**MM5**	**MM6**	**MM7**	**MM8**	**MM9**	**MM10**	**MM11**	**MM12**

Section 4-a. Household characteristics

	Main wage earner								Biological Father			Household		
Household ID	Who is the main wage earner in your family? Please indicate by using the HH member ID in section 1.	Age of the main wage earner?	Gender of the main wage earner? 1=Male 2=Female	Is the main wage earner engaged in **any business** or **wage labour (incl. farm labour)** in the last 12 months? **1=YES 0=NO**	What is the occupation of the main wage earner?	Biz Code	Number of months worked in the last 12 months	Annual income in the last 12 months in relation to H5 in ZMK	Is the father of this child the biological father? 1=Yes, 0=No[If No, skip H10-H11, and go to H12]	What is the height of biological father in CM?	What is the weight of biological father in kg?	Has this household ever received support from any health program by Government, NGO etc.? **1=YES 0=NO**	If Yes to H12, what type of support did this household receive? See Code below	Total size of owned land in hectares
ID	H1	H2	H3	H4	H5	H6	H7	H8	H9	H10	H11	H12	H13	H14

Code for H6:

1=Farmer
2=Trader
3= Marketer
4= Fisher man
5= Bricklayer
6= Ka ntemba
7= Carpenter
8= Stone crusher
10= Civil servant
11= Cleaner
12= Piece work
13=Others (specify)

Code for H13:
1= Mosquito net
2=Vitamin A supplement
3=Deworming
4=High Energy Protein Supplement (HEPS)
5= Plumpynnuts
6=Chlorine
7=Ready to Use Therapeutic Foods

Section 4-b. Consumption and Expenditure on Major Items (Non-Durable Goods) in the Past 12 Months

Instruction: *If the household does not purchase the item, ask how much the household would pay for the item per kg. ** EX0=23 – 42, ask only <u>total expenditure</u>. CHECK Unit code in code sheet in the last page.

**Consumed/Purchased Products**			Quantity consumed	Quantity purchased	Price per unit (Zmk)*	**Consumed/Purchased Products**			Total expenditure (Zmk)**
--During the last 7 days--	**EX0**	unit	**EX1**	**EX2**	**EX3**	***---During the last 30 days---***	**EX0**	unit	**EX4**
Staples						Sugar	23		
Maize grain	1					Salt	24		
Maize meal	2					Cooking oil/Palm oil	25		
Millet/Sorghum	3					Coffee/Tea	26		
Wheat flour	4					Drinks: Sodas, beer , Alcohol etc.	27		
Bread/any wheat products	5								
Rice	6					Tobacco/Cigarettes	28		
Fresh Cassava (Boiled/Roasted)	7					Restaurant Expense	29		
Processed Cassava (Flour/Chip)	8					Transportation	30		
Sweet potatoes	9					Cooking/Lighting fuel	31		
Irish potatoes	10					Soap/washing products	32		
Chicken	11					***--During the last 365 days--***			
Meats (any)	12					School fees, textbooks, etc	33		
Fish (Fresh/Dried)	13					Medical care	34		
Kapenta	14					Clothing/Shoes	35		
Chisense	15					***Contributions***			
Beans	16					Village Banking	36		
Ground nuts	17					Remittances to relatives	37		
Cow Peas	18					Churches/Mosques	38		
Vegetables (any)	19					Credit repayments	39		
Fruits (any)	20					Funeral Contribution	40		
Eggs (#number)	21					Contribution to other community functions	41		
Milk: liquid (litre)	22					Other local organizations	42		

Section 4-c. Household Assets

Instruction: A2-Ask "Suppose someone wants to buy your assets, at what price are you willing to sell them?"
Instruction: A3-Ask "Suppose someone has the exactly same assets as you, at what price are you willing to buy them?"

Asset		Number of assets currently owned	At what price, are you willing to sell this (these) asset(s) today? (Zmk)	At what price, are you willing to buy this (these) asset(s) today? (Zmk)	Number of assets **purchased in the past 12 months**
**ITEM**	**A0**	**A1**	**A2**	**A3**	**A4**
Durable Items					
Bicycle	1				
Radio	2				
Car Batteries	3				
TV	4				
Mobile Phones	5				
Sofa	6				
Tables	7				
Beds	8				
Mosquito nets	9				
Motorcycle	10				
Vehicles	11				
Tractor	12				
Fish pond	13				
Others (specify) :	14				

Section 4-d: Main House

Instruction: Answer based on interviewer's observation

MH1, What is the roofing material of the main house?

1=Grass/ crop material 2=Iron sheets 3=Tiles 6= Other ☐ specify___________________________________

MH2, What is the material of windows and doors (enumerators observation)

1=Glass/metallic ☐ 2= Wooden ☐ 3=grass/crop material ☐ 4= Other ☐ specify___________________________________

MH3, What is the main wall material of the major part of the house?

1=Mud ☐ 2=Bricks/Stone☐ 3=mud and pole ☐ 4=Grass/crop material 5=Iron sheets 6=Other specify____________________________

MH4, What is the floor material of the main house?

Section 4-d. Physical access to services

HP1: Has this household ever had electricity? 1=Yes, 0=No (If No, skip HP2 and go to the table) **HP1** ______________

HP2: Since which year has this household had electricity? **HP2** ______________

***If more than one transportation modes are used, select most frequently used mode.**
**** Ask the transportation mode, cost, and time to primary and secondary school where children of this household are currently attending.**
****If no member is currently attending school, ask the transportation mode, cost, and time to the nearest primary school and secondary school from the household.**

	Dry Season			Rainy Season		
	Typical transportation mode* Code	Transportation cost **(one way)** (Zmk)	Total travelling time **in minutes (one way)**	Typical transportation mode* Code	Transportation cost **(one way)** (Zmk)	Total travelling time **in minutes (one way)**
**ZID**	**Z1**	**Z2**	**Z3**	**Z4**	**Z5**	**Z6**
1. Primary school**						
2. Secondary school**						
3. Government hospital						
4. Rural Health Centre						
5. Mobile clinic						
6. Drinking water						
7. Market						

Code for Z1 and Z4
1=by foot
2=bicycle owned
3=motorbike owned
4=car owned
5= taxi
6=mini-bus
7= bus
8= other

Ethnic Group Code		Religion Code	Formal Education code	Unit Code
1 Bemba<br>2 Tonga<br>3 Chewa<br>4 Ngoni<br>5 Lamba<br>6 Lozi<br>7 Lenje<br>8 Chokwe<br>9 Tumbuka<br>10 Ila<br>11 Lunda<br>12 Luvale<br>13 Kaonde<br>14 Namwanga<br>15 Mbunda<br>16 Soli<br>17 Chewa<br>18 Nsenga<br>19 Toka leya<br>20 Bushi<br>21 Bisa<br>22 Ng'umbo<br>23 Shila<br>24 Bwile<br>25 Chishinga	26 Ushi<br>27 Swaka<br>28 Lala<br>29 Nsenga Luzi<br>30 Goba<br>31 Mfungwe<br>32 Mambwe<br>33 Lungu<br>34 Nkoya<br>35 Luchazi	1 No religion<br>2 Christian<br>3 Hindu<br>4 Muslim<br>5 Traditional<br>6 Other (specify)<br>……………………..<br>................................<br><br>**Marital Status**<br><br>1 Single<br>2 Monogamous<br>3 Polygamous<br>4 Widowed<br>5 Separated<br>6 divorced<br>7 Other (Specify)<br>……………………<br>……………………<br><br>**General codes**<br>-9=Not applicable<br>-8 = Don't know<br>-7 = Refuse to answer<br>-6 = Don't recall<br>-5 = Missing Data<br>1= Yes<br>0= No	0 Some primary<br>1 Primary 1<br>2 Primary 2<br>3 Primary 3<br>4 Primary 4<br>5 Primary 5<br>6 Primary 6<br>7 Primary 7/Junior 1<br>8 Secondary Grade 8,<br>9 Secondary Grade 9,<br>10 Secondary Grade 10,<br>11 Secondary Grade 11,<br>12 Secondary Grade 12,<br>13 Tertiary 1<br>14 Tertiary 2, Two-year collage completed<br>15 Tertiary 3<br>16 Tertiary 4, Four-year college completed.<br>17 Tertiary 5<br>18 Post-graduate and above<br>19 No formal education	-***Vegetable***<br>1=KG<br>2=UNIT<br>3=10kg bag<br>4=12.5kg bag<br>5=20kg bag<br>6=25kg bag<br>7=50kg bag<br>8=CRATE<br>9=BOX (banana)<br>10=PILE<br>11=BUNDLE<br>-***Other commodities***<br>1=KG<br>8=CRATE<br>12=10KG<br>13=15KG<br>14=20KG<br>15=25KG<br>16=30KG<br>17=10KG BAG UNSHELLED<br>18=10KG BAG SHELLED<br>19=25KG BAG UNSHELLED<br>20=25KG BAG SHELLED<br>21=90KG BAG UNSHELLED<br>22=90KG BAG SHELLED<br>23=TONE<br>24=2LITRE TIN<br>25=20LITRE TIN<br>26=5LITRE GALLON<br>27=20LITRE GALLON<br>28=CUP<br>29=MEDA<br>30=Ka Bp<br>31=LITRE<br>32=HEAD<br>33=TRAY<br>34=LIVE BIRD<br>35=BASIN<br>36=UMUTUNGO<br>37=DRIED SMALL FISH (KG)

Appendix 8: Dietary Diversity Questionnaire

Draft Ver.4.1

Dietary Diversity Record Survey (April 2015)
Alliance Forum Foundation, Japan (AFF) and Programme Against Malnutrition, Zambia (PAM)

Date of interview Date: ……….. Month: …….……Year: _________

Interviewed by: _____________________ Interviewer ID __________

Date checked:Date: ……….. Month: ………….Year: _________

Checked by: __________________ Officer ID __________

Date entered: Date: ……….Month: ………… Year: _________

Entered by: __________________ Officer ID __________

Household Head Name in 2015: __________________________

Main Respondent Name: _______________________________

HH member ID of main respondent: _____________

INSTRUCTION FOR INTERVIEWER:
Please read through the list of type of foods outlined in the table below and find out whether or not the household or any household member ate any of the listed food items under each group of food during the past seven days; Sunday to Saturday.
NOTE:
"Consider foods eaten by any member of the household and exclude foods purchased and eaten outside the home." If the response is;

a). Yes indicate one (1),
b). No, indicate zero (0),
c). Not applicable, indicate negative nine (-9),
d). Don't Know indicate negative eight (-8),
e). Refuse to answer indicate negative seven (-7),
f). Don't recall indicate negative six (-6) and
g). Missing data indicate negative five (-5.)

Example of foods in group1, groupe2,…, group7, respectively.

			**Child Dietary Diversity**							**Household member Dietary Diversity**						
**ID**	**Group**	**Name**	**Sun**	**Mon**	**Tue**	**Wed**	**Thu**	**Fri**	**Sat**	**Sun**	**Mon**	**Tue**	**Wed**	**Thu**	**Fri**	**Sat**
1	1. starchy staples (foods made from grain, roots, or tubers)	Nshima														
2		Bread														
3		Buns														
4		Fritter														
5		Scone														
6		Cassava														
7		Sweet Potato														
8		Rice														
9		Porridge														
10		fresh maize, samp														
11		noodles (spaghetti, macaroni, and other noodles)														
12		Maheu														
13		munkoyo														
14		Irish Potatoes														
15		other flour based pastry (samosa, pie, biscuits)														
16		Honey														
17		Sugar														
18		Cornflake														
**GROUP 2: Legumes**																
19	2. Legumes	Groundnuts														
20		Groundnut powder														
21		Beans														
22		Soya Pieces														
			**Child Dietary Diversity**							**Household member Dietary Diversity**						
**ID**	**Group**	**Name**	**Sun**	**Mon**	**Tue**	**Wed**	**Thu**	**Fri**	**Sat**	**Sun**	**Mon**	**Tue**	**Wed**	**Thu**	**Fri**	**Sat**

23		Soya Beans														
24		Soya powder														
25		Pumpkin seeds														
26		Soya milk														
27		Peanut butter														
28		Bambara nuts														
29		Sunflower seeds														
30		Cowpea														
**GROUP 3: Dairy (milk other than breast milk, cheese, or yogurt)**																
31	3. dairy (milk other than breast milk, cheese, or yogurt)	cow milk														
32		goat milk														
33		yoghurt														
34		yoghurt drink														
35		cheese														
36		sour milk														
37		super shake														
38		ice cream														
39		custard														
40		flavoured milk														
**GROUP 4: Meat, Poultry, Fish, or Eggs**																
41	4.meat, poultry, fish, or eggs	Beef														
42		chicken														
43		Pork														
44		Egg														
45		kapenta														
46		chisense														
47		fresh fish														
			**Child Dietary Diversity**							**Household member Dietary Diversity**						
**ID**	**Group**	**Name**	**Sun**	**Mon**	**Tue**	**Wed**	**Thu**	**Fri**	**Sat**	**Sun**	**Mon**	**Tue**	**Wed**	**Thu**	**Fri**	**Sat**
48		small fish														

49		dried fish														
50		offals														
51		other meat (duck, guinea fowl, pigeon, rabbit, hippo, game meat)														
52		Liver														
53		mbewa														
54		catapillar														
55		mince meat														
56		sausage and other processed meat (ham, jerky, becon)														
57		goat meat														
**GROUP 5: vitamin A-rich fruits and vegetables (pumpkin; red or yellow yams or squash; carrots or red sweet potatoes; green leafy vegetables; fruits such as mango, papaya, or other local vitamin A-rich fruits)**																
58	5. vitamin A-rich fruits and vegetables (pumpkin; red or yellow yams or squash; carrots or red sweet potatoes; green leafy vegetables; fruits such as	Orange Maize														
59		Orange Sweet Potato														
60		pumpkin														
61		carrot														
62		rape														
63		Chinese														
64		pumpkin leaves														
65		cowpea leaves														
66		Okra														
67		dried pumpkin leaves														
			**Child Dietary Diversity**							**Household member Dietary Diversity**						
**ID**		**Name**	**Sun**	**Mon**	**Tue**	**Wed**	**Thu**	**Fri**	**Sat**	**Sun**	**Mon**	**Tue**	**Wed**	**Thu**	**Fri**	**Sat**
68		dried cowpea leaves														
69		dried okra														
70		spinach														
71		green pepper														
72		okra leaves														
73		cassava leaves														

74	mango, papaya, or other local vitamin A-rich fruits)	dried cassava leaves														
75		Bondwe (amaranthus)														
76		sweet potato leaves														
77		red guava														
78		mango														
79		Papaya														
**GROUP 6: Other fruits and vegetables (or fruit juices)**																
80		egg plant														
81		Impwa														
82		Avocado														
83		Tomato														
84		cucumber														
85		Onion														
86		Cabbage														
			**Child Dietary Diversity**							**Household member Dietary Diversity**						
**ID**	6.other fruits and vegetables (or fruit juices)	**Name**	**Sun**	**Mon**	**Tue**	**Wed**	**Thu**	**Fri**	**Sat**	**Sun**	**Mon**	**Tue**	**Wed**	**Thu**	**Fri**	**Sat**
87		Pineapple														
88		grapes														
89		Mulberry														
90		water melon														
91		fruit juice														
92		white guava														
93		Apple														
94		Orange														
95		lemon														
96		fruit juice 100%														
97		Banana														
98		mushroom														

99		wild fruits														
100		Masau														
**GROUP 7: Oil, fat, or butter**																
101	7. oil, fat, or butter	Cooking oil														
102		Butter or margarine														
103		Red palm oil														

Dietary Diversity Record: Summary

		Did this child eat foods from Group 1, Group 2, ---, Group 7, last Monday to Sunday, respectively?<br>1=YES, 0=NO						
House hold ID		Group1: Starchy staples	Group2: Legumes	Group3: Dairy	Group4: meat, poultry, fish, or eggs	Group5: vitamin A-rich fruits and vegetables	Group6: Other fruits &vegetables	Group7: Oil, fat, or butter
**ID**		**DDR1**	**DDR2**	**DDR3**	**DDR4**	**DDR5**	**DDR6**	**DDR7**
	**Sunday**	**1**	**0**	**0**	**0**	**1**	**0**	**0**
	**Monday**	**1**	**0**	**0**	**1**	**1**	**0**	**1**
	**Tuesday**	**1**	**0**	**0**	**0**	**1**	**0**	**0**
	**Wednesday**	**1**	**0**	**0**	**0**	**1**	**0**	**0**
	**Thursday**	**1**	**0**	**0**	**0**	**1**	**0**	**0**
	**Friday**	**1**	**0**	**0**	**0**	**1**	**0**	**0**
	**Saturday**	**1**	**0**	**0**	**0**	**1**	**0**	**0**
	**How many days, did this target child eat foods from Group1, Group2, …, Group7? [0-7]**	**7**	**0**	**0**	**1**	**7**	**0**	**1**

Dietary Diversity Record: For Data Entry

Household ID	Did this child eat foods from Group 1, Group 2, Group 3, Group4, Group 5, Group 6, Group 7, respectively for 3 or more days in previous 7 days?<br><br>1=Yes<br>0=No							During last 7 days, how many groups did this child eat foods from Group 1, Group 2, Group 3, Group4, Group 5, Group 6, Group 7, respectively?<br>(Interviewer's calculation: Sum up the value in DDR1, DDR2,…., DDR7)
	Group1: Starchy staples	Group2: Legumes	Group3: Dairy	Group4: meat, poultry, fish, or eggs	Group5: vitamin A-rich fruits and vegetables	Group6: Other fruits &vegetables	Group7: Oil, fat, or butter	Score (Takes the value between 0 and 7)
**ID**	**DDR8**	**DDR9**	**DDR10**	**DDR11**	**DDR12**	**DDR13**	**DDR14**	**DDR15**
	**1**	**0**	**0**	**0**	**1**	**0**	**0**	**2**

Dietary Diversity Record: *Quantity*

Household ID	During last 7days, how many times did a child eats food other than breast milk per day						
	Sunday	Monday	Tuesday	Wednesday	Thursday	Friday	Saturday
**ID**	**DDR16**	**DDR17**	**DDR18**	**DDR19**	**DDR20**	**DDR21**	**DDR22**
	**3**	**3**	**2**	**2**	**3**	**2**	**2**

Appendix 8: Monthly Morbidity Questionnaire

Draft Ver.4.1

Monthly Morbidity Record Survey (April 2015)

Alliance Forum Foundation, Japan (AFF) and Program Against Malnutrition, Zambia (PAM)

Date of interview Date: ……….. Month: …………….Year: _________

Interviewed by: _____________________ Interviewer ID ______

Date checked: Date: ……….. Month: ………… Year: _________

Checked by: __________________ Officer ID ______

Date entered: Date: …………..Month: …………Year: _________

Entered by: __________________ Officer ID ______

Household ID: ___________________

Household Head Name in 2015 ___________________________

Main Respondent Name ________________________________ HH member ID of main respondent: ______________

Instruction for interviewer
Please ask questions to the mother.

Use following general codes if applicable

-9=Not applicable	-7 = Refuse to answer	-5 = Missing Data
-8 = Don't know	-6 = Don't recall	1= Yes, 0= No

Section 1. Child's Morbidity Monthly Record

Household ID	Pneumonia		Measles & Malaria				Diarrhoea		Hospital	
	Has (NAME of the child) had a cough at any time in the last 4 weeks? 1=Yes 0=No [If the answer to CM1 is No, skipCM2 and go to **CM3**]	If "Yes" to CM1, did he/she breathe faster than usual with short, rapid breaths or have difficulty breathing? 1=Yes 0=No	Has (NAME of the child) had a high fever in the last 4 weeks? 1=Yes 0=No [If the answer to CM3 is No, skip CM4-CM6 and go to **CM7**]	If yes to CM3, did he/she has rash on his /her body after fever? 1=Yes 0=No	If yes to CM3, did he/she has a fever with a chill, shaking and nausea? 1=Yes 0=No	If yes to CM3, did he/she have swings of low temperature and high temperature? 1=Yes 0=No	Has (NAME of the child) had *diarrhoea at any time in the last 4 weeks? *have loose stools more than 3 times per 24hours 1=Yes 0=No [If the answer to CM7 is No, skip CM8 and go to **CM9**]	If yes, how many days in a row has he/she had a diarrhea?	If your child had any of the symptoms listed in CM1-CM8, did you take him/her to the hospital? 1=Yes 0=No [If the answer to CM9 is No, skip CM10 and go to **MM1**]	If yes to CM9, what was he/she diagnosed with? **<u>LIST ALL & USE Code</u>**
**ID**	**CM1**	**CM2**	**CM3**	**CM4**	**CM5**	**CM6**	**CM7**	**CM8**	**CM9**	**CM10**

Section 2. Mother's Morbidity Monthly record

Household ID	Pneumonia		Measles & Malaria				Diarrhoea		Hospital	
	Have you had a cough at any time in the last 4 weeks? 1=Yes 0=No [If the answer to MM1 is No, skip MM2 and go to **MM3**]	If "Yes" to MM1, did you breathe faster than usual with short, rapid breaths or have difficulty breathing? 1=Yes 0=No	Have you had a high fever in the last 4 weeks? 1=Yes 0=No [If the answer to MM3 is No, skip MM4-MM6 and go to **MM7**]	If yes to MM3, did you have rash on your body after fever? 1=Yes 0=No	If yes to MM3, did you have a fever with a chill, shaking and nausea? 1=Yes 0=No	If yes to MM3, did you have swings of low temperature and high temperature? 1=Yes 0=No	Have you had *diarrhoea at any time in the last 4 weeks? *have loose stools more than 3 times per 24hours 1=Yes 0=No [If the answer to MM7 is No, skip MM8 and go to **MM9**]	If yes, how many days in a row did you have diarrhea?	If you had any of the symptoms listed in MM1-MM8, did you go to the hospital? 1=Yes 0=No [skip MM10]	If yes to MM9, what were you diagnosed with? **<u>Code Sheet</u>**
**ID**	**MM1**	**MM2**	**MM3**	**MM4**	**MM5**	**MM6**	**MM7**	**MM8**	**MM9**	**MM10**

Code for CM10&MM10:

1= pneumonia
2= measles
3= malaria
4= diarrhoea
5= fever
6= others (specify)

Monthly Compliance Record Survey (April 2015)

Instruction: Ask the mother of the target child “Did you feed the distributed porridge (with spirulina or without spirulina) to the target child last Monday, Tuesday, Wednesday, Thursday, Friday, Saturday, and Sunday, respectively.

Household ID	Compliance<br>Did you feed the child the distributed porridge last …<br>1=Yes<br>0=No							How many days did the mother feed the child the distributed porridge during the last 7 days?<br>(Interviewer’s calculation:<br>Sum up the value in MCR1, MCR2,…., MCR7)
	Sunday	Monday	Tuesday	Wednesday	Thursday	Friday	Saturday	Score<br>(Takes the value between 0 and 7)
**ID**	**MCR1**	**MCR2**	**MCR3**	**MCR4**	**MCR5**	**MCR6**	**MCR7**	**MCR8**

Appendix 9: Letters of Approval

Care International in Zambia
Plot 9, Chitemwiko Close, Kabulonga
P. O. Box 36238
Lusaka, Zambia

Tel: +260 211 267950-4, 58-9
Fax: +260 211 267956-7
E-mail: info@carezam.org
Website:http://www.careinternational.org

2nd March 2015AD

The Chairperson,
Biomedical Research Ethics Committee,
Ridgeway Campus,
P.O. Box 50110,
Lusaka.
ZAMBIA

Dear Sir/Madam

Re: SUPPORT FOR SPIRULINA PRODUCTION AND UTILIZATION RESEARCH IN LUAPULA PROVINCE

CARE Zambia has been collaborating with Programme Against Malnutrition in the area of promoting research in foods with potential high impact on reducing stunting in the population and especially among children under two years of age. PAM has identified Spirulina as one such food additive which has very high content of protein (approximately 67%) and therefore potentially very useful in the fight against malnutrition. They have prepared a research protocol which has been shared with CARE Zambia. Based on the research protocol CARE Zambia decided to partner with them. In partnering with PAM CARE Zambia has also provided funding to PAM for the research which will take place in Luapula Province specifically Mansa and Samfya Districts.

Given the importance that CARE Zambia has given to the fight against malnutrition any assistance that can be given to PAM to enable them undertake this important research will be greatly appreciated.

Yours sincerely,

Dennis O'Brien
COUNTRY DIRECTOR

Cc PAM

CARE Vision: We seek a world of hope, tolerance and social justice, where poverty has been overcome and people live in dignity and security.

THE NATIONAL
FOOD AND NUTRITION COMMISSION

Lumumba Rd. P.O. Box 32669, Lusaka Tel 0211 227803 / 0211 227804, Fax: 0211 221426, Email: nfnc@zamtel.zm

Your Ref. Date:

Our Ref.

March 02, 2015

The Chairperson
Biomedical Research Ethics Committee
Ridgeway Campus
P.O Box 50110
Lusaka

Dear Sir/ Madam,

RE: Support of Spirulina Promotion in Luapula Province

The National Food and Nutrition Commission (NFNC) has closely collaborated with the Programme Against Malnutrition (PAM) in the institution's effort to eliminate malnutrition in Zambia. The spirulina effectiveness test conducted in Kanakantapa, Chongwe district revealed positive and encouraging results such that NFNC is eager to see the activities scaled up in Luapula province where the rate of stunting is high at 49% (LCMS, 2010).

The NFNC is keen on promoting strategies that will help eliminate malnutrition in Zambia in line with government's strategic initiative of the First 1000 Most Critical Days. The Commission highly supports the effort by the Programme Against Malnutrition to scale up spirulina activities in Luapula province under the Promotion of Spirulina Production and Utilization project.

It is the Commission's considered view that the results of the Luapula study will strengthen the rationale for approving spirulina as food for promoting good growth and health in Zambia.

Your favourable consideration and approved of the protocol will be greatly appreciated as PAM in collaboration with key stakeholders makes concerted efforts to eliminate malnutrition.

All communications should be addressed to the Executive Director

Yours faithfully,

Robinah Mulenga - Kwofie (Mrs)
Executive Director

THE UNIVERSITY OF ZAMBIA
SCHOOL OF AGRICULTURAL SCIENCES
DEAN'S OFFICE

P.O. BOX 32379, Lusaka, Zambia
Telephone: 250587
Fax: 250587/255655/253952

Telegrams: UNZA LUSAKA
Telex: ZA 44370, fax
Tel. +260 250 587

dean-agric@unza.zm, mmwala@unza.zm,
mmwala@yahoo.com

The Chairman
Biomedical Ethics Committee
University of Zambia
P.O. Box 32379
LUSAKA

26th February 2015

Dear Sir,

SUPPORT LETTER: SPIRULLINA RODUCTION BY SPIRULLINA SUN PROJECT

The School of Agricultural Sciences hosts a project jointly run with Programme Against Malnutrition (PAM) which has gone through a pilot phase. There is an intention to upscale this activity in Luapula Province by PAM under the auspices of the Promoting Spirullina Production and Utilization (PSPU) project.

This action is fully supported by the School of Agricultural Sciences, who will continue participating in the project, realizing the value of the outcome towards improved nutrition.

Your favourable consideration of the application for approval from your committee will be most appreciated.

Mick S. Mwala, PhD
DEAN – SCHOOL OF AGRICULTUTAL SCIENCES

cc Acting Executive Director Programme Against Malnutrition (PAM)

Appendix 10: Investigator CV

CURRICULUM VITAE	MAUREEN CHITUNDU
**Name**	Maureen Mukabo Chitundu
**Marital Status:**	Married
**Nationality:**	Zambian
**Profession:**	Nutritionist

Key Qualifications:

Mrs. Chitundu has 27 years of experience in smallholder agriculture and rural development with focus on food security, food processing and nutrition; with 10 years middle management capacity dealing with programme implementation at PAM. She has been involved in designing and managing projects implemented by government and PAM i.e. (a) Luapula Livelihood and Food Security Programme (FINNIDA) (b) Enhancing Household Food Security and Nutrition in the Luapula Valley (FAO) (c) Smallholder Access to Processing, Extension and Seeds (SHAPES) Project (Sida/NORAD) (d) Food Security Pack (Government) (e) Enhancing Food Security in Cassava Based Farming Systems (FAO) (f) Processing of High Grade Cassava Products (Embassy of Japan).

Mrs. Chitundu is familiar with the participatory approach to rural development having worked with projects funded by different cooperating partners. Mrs. Chitundu is a member of the Zambia Cassava Sub-sector Strategy Committee (participated in Country Cassava Strategy Development), Task Force on Food Based Approaches to Eliminating Micro Nutrient Deficiencies in Zambia and HarvestPlus Orange Maize Steering Committee.

Education:

**2015**	Candidate Bachelor of Arts Degree in Development Studies with Zambian Open University, Lusaka, Zambia
**2003**	Diploma in Food and Nutrition Security **-** International Agriculture Centre, the Netherlands.
**1996**	Postgraduate Diploma in Crop Storage and Processing - Cranfield University, UK
**1986**	Diploma in Nutrition, NRDC, Zambia
**1982**	School Certificate

Employment Record:

**2012-todate**	Acting Executive Director (PAM) – Strategic management of the institution, development of project proposals, facilitating effective implementation, monitoring and evaluation of projects, networking, report writing and information dissemination to all stakeholders.
**2010-2012**	Senior Programme Officer (PAM) – Facilitating effective

implementation of projects, coordinating NGOs and CBOs in respective provinces to facilitate networking and enhance collaboration among participating institutions, development of monitoring and evaluation instruments and conducting timely, effective and efficient monitoring and evaluation exercises of projects, training, report writing and information dissemination to all stakeholders.

2000-2010 Food Processing Specialist (PAM) - Design, coordination and implementation of food processing, storage and nutrition activities focused at household level with a bias towards under five children i.e. training of line ministry officers and beneficiaries, production of training manuals, facilitating product development and establishment of cottage industries, writing project proposals, networking with institutions engaged in food processing and nutrition. Monitoring and evaluating activities and report writing

1997-2000 Chief Technical Research Assistant. MAFF - Farming Systems and Social Sciences Team. Supervised and co-ordinated implementation of trials and studies, supervised adaptation of post harvest technologies, organized and facilitated farmer and staff food processing and nutrition workshops, report writing, monitoring and evaluation of activities

1991- 1996 Nutritionist (MAFF). Farming Systems and Social Sciences Team. Implementing and supervising adaptation of postharvest technologies and conducting food processing, storage and nutrition workshops for extension staff and small-scale farmers. Monitoring activities and report writing

1986 – 1990 Practical Instructor and Boarding Mistress – Kalulushi Farm College (MAFF). Designing, training and evaluating the nutrition training Programme. General counselling and facilitating provision of conducive learning environment

---

Record of Major Projects:

**2012 to date**	**Programme Against Malnutrition**
Title	Acting Executive Director
Project Name	Empowering Women in Senanga and Gwembe Districts through Agricultural Support Project by PAM (2 ½ years). Designing and coordinating implementation of project in Conservation Farming Unit and COMACO. Facilitate training of officers and farmers in production, nutrition and agribusiness. Networking, monitoring and evaluating project activities; report writing and dissemination of information to stakeholders

Operational Area	Gwembe and Senanga districts

**2011**	**Programme Against Malnutrition**
Title	Senior Programme Officer
Project Name	Spirulina Pilot Project (9 months) by PAM. Design, implementation and coordinating the research project in collaboration with Alliance Forum Foundation. Assessing the effectiveness of spirulina in reducing malnutrition in under five children. Training of government officers and child growth promoters in nutrition, accurate body measurements and data collection. Monitoring and supervising field activities; facilitating data collection, analysis and dissemination of findings to stakeholders
Operational Area	Chongwe district

**2010**	**Ministry of Community Development and Social Services**
Title	Food Processing Specialist
Project Name	Food Security Pack Project implemented by PAM (10 years). Design, coordinating and training of government officers and NGOs in food storage, processing, utilization and, nutrition and HIV/AIDS. Facilitated product development for example weaning blends with NISIR and food industry, WFP installation of milling plant in Mansa and institution of quality control measures in cassava processing at community and Milling plant level; growth monitoring of under five children in collaboration with the MoH and NFNC. Monitoring and supervising field activities.<br>Managed and coordinated FSP project implementation, supervision and monitoring in Lusaka province i.e. input procurement and distribution, supervision and monitoring, financial management, report writing and networking.
Operational Area	All 72 districts

**2009**	**Food and Agriculture Organization (FAO)**
Title	Food Processing Specialist
Project Name	Enhancing Food Security in Cassava Based Farming Systems (2 years). Planning, coordinating and supervising field cassava production and postharvest activities.

	Conducting processing and value addition workshops for officers and cassava processors. Facilitated construction of storage structures and market linkages
Operational Areas	Central and Luapula provinces

**2009**	**Embassy of Japan**
Title	Food Processing Specialist
Project Name	Processing of High Grade Cassava Products (one year). Managed and coordinated construction of Kanakantapa Cassava Processing Centre. Facilitated market linkages and cassava product promotions. Organized and facilitated training in technical skills i.e. processing and storage, equipment operations, group dynamics and leadership and, entrepreneurship skills.
Operational Areas	Chongwe district

**2008 to 2010**	**Swedish Development Agency (Sida)**
Title	Food Processing Specialist
Project Name	Cassava Transformation in Southern Africa (CATISA). An action-oriented research consortium spanning the research-policy nexus. Comprise researchers and practitioners from 4 countries including private sector, NGOs, IITA, NRIs. The objective was to accelerate cassava commercialization through comparative cross-country analysis and cross-fertilization, understand how and why commercialization processes differ across countries and zones and share cassava processing technology and food safety procedures across countries
Operational Areas	Tanzania, Malawi, Mozambique and Zambia. Collaborates with Ghana, Denmark, Nigeria, Sweden and USA.

**2004**	**Swedish International Development Agency (Sida)**
Title	Food Processing Specialist
Project Name	Smallholder Access to Processing, Extension and Seeds project (3 years)<br>Design, coordinate and train government officers and NGOs in food storage, processing, utilization and nutrition. Facilitated procurement of processing equipment and establishment of income generating activities among women groups. Monitoring, backstopping and supervising field activities

Operational Areas	Western, Central, Southern and Eastern provinces

**2000**	**Finnish International Development Agency (FINNIDA)**
Title	Sub Component Head - Nutrition
Project Name	Luapula Livelihood and Food Security Programme (2 years). Planning and organizing food processing, storage and nutrition workshops for farmers and officers. Networking and assessing effectiveness of cassava batch dryers.
Operational Areas	Luapula province

Other Relevant Information Regarding the Expertise:

2010: Part of a three man consultancy team that conducted a study particularly to determine the benefits that accrue to small holder farmers as a result of the developments that were taking place in the cassava value chain.

2007: Part of the CATISA Consortium that conducted a baseline survey on cassava processing technologies, food Safety and utilization methods in Zambia and Malawi. The findings provided a basis for strengthening on-going cassava activities by various partners.

1997: Part of 15 member provincial team that conducted situation analysis on health and nutrition in schools of the Luapula valley. Findings formed basis for incorporating health, food security and nutrition in Zambian primary education curriculum as well as adapting the FAO handbook for local level nutrition education.

May 1994: Part of a consultancy team that produced the Indicative Development Plan for Luapula Province. Contributed chapter on Nutrition

Other Courses and Workshops Attended:

Southern Africa Region CAADP Nutrition capacity development workshop, mainstreaming nutrition within CAADP investment plans and processes. Organized by NEPAD and African Union, Botswana, Gaborone 9th – 14th September 2013

AfricaLead Champions for change training workshop, by the US Agency for International Development with contributions from Continuing Education, University of Pretoria, South Africa. Protea Hotel Chisamba, Zambia - 16th - 20th April 2012

Food and Nutrition Security and HIV/AIDS course, by International Agriculture Centre, Wageningen in collaboration with Natural Resources Development College, Lusaka Zambia – 17th – 30th April 2005.

Nutritional Care and Support for People Living with HIV/AIDS Course, by the Institute of Food, Nutrition and Family Sciences in collaboration with IAC, University of Zimbabwe, Harare, Zimbabwe, 10th – 21st February 2003.

Agro-Enterprise Development Training Course for government and NGO officers, by FOODNET, Botanical Beach Hotel, Entebbe, Uganda, 2nd – 11th May 2001.

Nutrition Orientation Workshop on Use of Training Guides for Luapula Province by FAO, Mansa Teacher's Training College, Mansa, Zambia, 27th June – 1st July 1994.

Brainstorming Workshop on streamlining development priorities for Luapula province, Luapula Rural Development Programme (LRDP)/FINNIDA, Mansa, Zambia, 19th – 21st January 1994

Computer Skills

Microsoft software: Windows 97 – 2003, 2007, 2010, MS Office (Word, Excel, Power Point)

Language:

**Languages**	**Spoken**	**Writing**	**Reading**
English	Good	Good	Good
Tonga	Good	Fair	Fair
Bemba	Good	Good	Good
Nyanja	Fair	-	Fair

Publications:

1. Cassava commercialization in Southeastern Africa – 2012
2. Establishing the viability of generating marketable surpluses of cassava – An assessment of domestic market opportunities with reference to the cassava value chain in selected regions in Zambia, Final Draft 2012
3. Chemical safety of cassava products in regions adopting cassava production and processing – experience from Southern Africa, 2010
4. Small holder cassava value chain asset survey preliminary report, 2010
5. Cassava Production, Processing and Utilization in Zambia, 2009
6. Cassava/Wheat Fritters an Innovation for Increased Profits, 2007
7. A value chain task force approach for managing private –public partnerships: Zambia's task force on acceleration of cassava utilization, 2006
8. Sweet Potato Processing Guide, PAM 2004
9. Food Processing and Storage Training of Trainers' Manuals; PAM - 2001

Contact **Official**

Address: Programme Against Malnutrition
P.O. Box 30599, Lusaka, Zambia.
Tel: 260-1-235941/2
Fax: 235939

E-mail: chitundu.maureen@gmail.com

Employment Referees

Ms. Doris Musonda
Lead Consultant, RuralNet Associates Ltd, Plot No. 6465, Libala Road, Kalundu
P. O. Box 51311, Lusaka, Zambia.
E-mail: dorismusonda@gmail.com

Mr. Ronald Msoni
Project Agronomist, Conservation Agriculture Scaling UP (CASU)
Food and Agriculture Organization, House No 5, Addis Ababa Drive
P.O. Box 30563, Ridgeway, Lusaka, Zambia
Mobile 260-977-751263

E-mail: ronaldmsoni@gmail.com

Professional Referees

Ms Dorothy Nthani
Senior Lecturer/Nutrition, Natural Resources Development College
P.O. Box 310099, **Lusaka**
Mobile: 0955 45 88 24

E-mail: dnthani@yahoo.com

Dr. Steven Haggblade
Professor, International Development, Department of Agricultural Economics
Michigan State University (MSU), East Lansing, MI 48824, **USA.**

E-mail: blade@msu.edu

CURRICULUM VITEA -2015

C/o Programme Against Malnutrition (PAM), Plot 178, Parirenyatwa Road, Fairview, Lusaka
Tel #: 235940/41

E-mail: chimuze2@yahoo.com, Cell: 0963856064 or 097623289,

PERSONAL DETAILS

NAME :	ALEXANDER CHISHALA MWAPE
NATIONALITY :	ZAMBIAN
NRC No.:	200443/31/1

ACADEMIC QUALIFICATIONS

PERIOD	INSTITUTION	FIELD OF STUDY	ATTAINMENT
1991 - 1994	Mansa Secondary school	O' Level GCE	School certificate

PROFFESSIONAL QUALIFICATIONS

PERIOD	INSTITUTION	FIELD OF STUDY	ATTAINMENT
1998- 2003	Natural Resources Development College **(NRDC)**	Food and Nutrition	Diploma in Food and Nutrition
Feb – April 2006	International Agriculture Centre ,**WAGENINGEN**	Food and Nutrition Security	Postgraduate Diploma
2008 -2012	UNIVERSITY OF ZAMBIA Great East Road Campus	Food Science and Technology	BSC IN FST
June – August 2013	UNIVERSITY OF ZAMBIA Great East Road Campus	participatory monitoring and Evaluation for projects/childhood Programmes	Certificate

WORKSHOPS AND SHORT COURSES ATTENDED

PERIOD	WORKSHOPS / SHORT COURSE
15TH - 19th September 2003	Training on exclusive Breast feeding, HIV and Infant Feeding
20th December 2003-20th January 2004	Computer training in Microsoft word, Excel and power point
18th - 27th November 2004	Management of severely Malnourished children in hospital
13th February -3rd march 2006	Key concepts and current issues in nutrition
6th -17 March 2006	Food and nutrition security in the context of HIV/AIDS
20th – 24th March 2006	International seminar on right based approaches for food.
27th March -14th April 2006	Monitoring & Evaluation of food and nutrition security policies and Programmes
17th -28th April 2006	Nutrition Communication and promotion
18th – 23rd December 2006	Management of severely malnourished children in hospital

WORKING EXPERIENCE

PERIOD	INSTITUTION	POSITION	Duty Station
June 2004 – June 2005	Lusaka District Health Management Team **(LDHMT)**	Nutritionist	Matero Reference Health Centre , Matero
July 2005 to date	Gwembe Community District Medical Office(GCDMO)	Nutritionist	Gwembe Community District Medical Office, Gwembe

TASKS AND RESPONSIBILITIES: LDHMT

1. Facilitate in the development of the nutrition component of the health centre action plan
2. Conduct nutrition training for health workers and other community health workers at the health centre.
3. Manage food aid according to the health center guidelines.

4. Conduct monitoring and evaluation of nutrition related programmes being implemented at the health centre and advice on matters concerning nutrition.

TASKS AND RESPONSIBILITIES CURRENTLY: GCDMO

1. Plan district nutrition interventions in line with the identified community problems.
2. Maintain up to date information /data base on nutrition status of people especially that of vulnerable groups.
3. Identify sub-populations at risk of nutrition problems and advise / explore appropriate action/ interventions.
4. Facilitate in the development of the nutrition component of the district action plan
5. Conduct nutrition training for health workers and other community health workers.
6. Manage food aid according to the district guidelines.
7. Conduct monitoring and evaluation of nutrition related programmes being implemented in the district and advice on matters concerning nutrition.
8. Plan, conduct and evaluate dietary studies.

CO-ORDINATION

- Link person between **GCDMO** and other organizations dealing in food and nutrition aspects in the area and overseer of nutrition activities carried out by nutrition groups in the district and also build partnership with other stake holders.
- Establish and maintain contact with the nutrition specialist at ministry of health and other stake holders.

OTHER ACTIVITIES (ELECTROL COMMISSION OF ZAMBIA)

- Conducted the September 2006 tripartite general elections as a Polling Assistant based at Luumbo basic school in Luumbo Gwembe district.
- Conducted the October 2010 tripartite general elections as a Presiding Officer based at Sompani Middle basic school in Munyumbwe Gwembe district.

HOBBIES

- Traveling
- Surfing the internet
- Watching movies and listening to music
- Cooking nice dishes
- Game viewing

REFEREES

Dr Mutale Chimutete<br>District Community Medical officer<br>Gwembe Community District Medical Office<br>P.O Box 34,<br>Gwembe.<br>Tel #03240001	Miss Nthani,<br>Head of Department (Nutrition Department)<br>Natural Resources Development College<br>P.O Box 310099, Chelstone<br>Lusaka.<br>Tel # 01283613
Mr K.Mtambo<br>District Planner,<br>Gwembe Community District Medical Office<br>P.O Box 34,<br>Gwembe.<br>Tel #03240001	Mr R. Mapulanga<br>District Aids Co-ordination Advisor<br>Gwembe District Aids Task Force (DATF)<br>P.O Box 1,<br>Gwembe.<br>Cell # 097525840

Yumiko Ota

Nihonbashi-Hongokucho 4-4-20, Mitsui 2nd Annex 7F, Chuo-ku, Tokyo 103-0021, Japan・+81-362252795・
y-ota@allianceforum.org

EMPLOYMENT

Alliance Forum Foundation (AFF) June 2012-Current
Deputy Programme Officer, Tokyo, Japan

- Managed a project to improve nutrition among children under five years of age in Zambia by organizing public and private stakeholders in Zambia and Japan, conducting market and consumer research, developing a research partnership with University of Zambia, overseeing two Japanese and five local staff, reporting to donors, and keeping financial records.
- Performed marketing research on high-nutrition food for maternal and lactating mothers and children in rural and urban areas of Bangladesh for a Japanese corporate client through household survey and pilot sales.
- Identified potential business seeds in Asia and Africa, summarized in potential project profiles, conducted due diligence, and successfully received funding of $1.2 million in total by submitting proposals to JICA.
- Fundraised for the nutrition programme in Zambia by communicating and proposing partnerships with CSR divisions at Japanese corporations, and successfully increased the partnerships from one company to five companies.
- Presented the progress in the nutrition programme in Zambia at seminars to increase recognition of the programme, and to recruit student and professional supporters to expand the programme activity.
- Networked with over fifty Japanese corporations by organizing networking seminars three times a year to identify potential clients, and increase the presence of Alliance Forum Foundation as a partner among Japanese corporate society.
- Provided business advisory service to Japanese corporate clients on their projects in Bangladesh and Kenya by managing Japanese and local staff.
- Analyzed over 200 household and customer data, and proposed marketing strategies to Japanese corporate clients.
- Negotiated with potential local partners (corporate and non-profit organizations) on signing business agreements for clients.

Japan Development Institute (JDI) February 2007-August 2010
Research Assistant, Tokyo, Japan

- Analyzed social, economical, and environmental impacts of development projects to local areas in Laos, Cambodia, Viet Nam, Indonesia, India and Tanzania by conducting field research, interviews and data collection/analysis, and summarizing into reports.
- Reviewed literatures, collected, sorted, and analyzed data sets, prepared reports, arranged meetings with governmental officials to assist research activities in Africa, Middle East, and South East Asia.
- Prepared project proposals, and took responsibility of accounting and human resource tasks of projects.

INTERNSHIPS

Kounkuey Design Initiative (KDI) July-August 2011
Community Organizer Intern, Nairobi, Kenya

- Worked as a community organizer by managing two local interns at three different sites in Kibera slum.
- Taught accounting and business management skills (how to plan expenses, how to manage inventory, and how to generate profit) to six community groups running small enterprises.
- Conducted a cost-benefit analysis for two small enterprises, and created business plans together with the community members to improve income generation activities.
- Assisted a foundation of a bakery run by community members at site 2.
- Assisted trainers to conduct business communication training to community members using computers.

- Conducted business needs assessment survey at a new project site and hold community meetings to identify new community business to support KDI's public space design project.

United Nations Centre for Regional Development (UNCRD) Africa Office May-August 2006
Intern, Nairobi, Kenya
- Interviewed about 10 residents joining local self-help group and local NGOs, and created a report on the impact of local initiative projects to the level of community building and improvement at slums in Nairobi.
- Worked in a team and reported the condition of Internally Displaced People in Northern Kenya and submitted to United Nations High Commission of Refugees (UNHCR) to create a joint project proposal.

Community Development Commission (CDC) of LA County January-May 2006
Intern, Monterey Park, CA
- Interviewed participants and county staffs to evaluate the effect of the community leaders training project.
- Prepared a report and conducted a presentation on the outcome of the evaluation to CDC and community leaders.
- Assisted CDC staffs to organize an empowerment and training workshops for community leaders.

EDUCATION
University of California, Los Angeles (UCLA), Los Angeles, CA
Master of Urban and Regional Planning June 2012
Emphasis: Housing and Community Economic Development GPA: 4.0

University of Southern California (USC), Los Angeles, CA
Bachelor of Science in Public Policy, Management, and Planning December 2006
Emphasis: Urban Planning and Development Major GPA: 3.7

AWARDS
Department of Urban and Regional Planning Faculty Award (UCLA) Spring 2012
W.K. Kellogg Foundation Social Justice Student Fellowships (UCLA) Fall 2011
Joint Japan / World Bank Scholarship Program (World Bank) Fall 2011
Vanessa Dingley Fellowship Award (UCLA) Spring 2011
Community Development Commission Exemplary Service Award (CDC-LA) Spring 2006
USC Leadership Award (USC) Spring 2005

TRAINING
Human Subjects Ethics and Regulations (UCLA Office of Research Administration) November 2011

SKILLS
Language: French (intermediary)
Computer: Microsoft Word, Excel, Power Point, Adobe Photoshop, GIS Desktop 10.0, SPPS

EXPERIENCES IN BUSINSS DEVELOPMENT PROJECTS
1) **Decentralized Bio Energy Supply Chain Development Study in Lao, PDR**
September 2007-March 2008
Socio-Environmental Impact Analyst, Bokeo, Vientiane, and Savannakhet, Lao PDR
- Researched on the socio-economic condition of farmers in three major cities in Laos (Bokeo, Vientiane, and Savannakhet).
- Analyzed the social (use of bioenergy to generate electricity) and economic (production of cash crops) impacts of introducing bio-energy to rural communities in Laos.

2) **Mtwara Port and Economic Development Zone (EDZ) Development Plan**
July 2008-March 2009
Socio-Economic Impact Analyst, Dar es Salaam and Mtwara, Tanzania

- Analyzed and summarized the socio-economic impact of expanding a port in Mtwara, and exporting the natural resources in the Mtwara Corridor.
- Analyzed and summarized the economical impact to the Mtwara region, Tanzania, and neighbor countries of having a more integrated transportation by developing the Mtwara Corridor.
- Analyzed the negative impact of relocating households in the project area of port expansion, and proposed a compensation plan a government can implement to minimize the negative impacts.

3) **Feasibility Study on the Integrated Waste Management System in Gujarat, India**
July 2009-March 2010
Socio-Environmental Impact Analyst, New Delhi, Ahmedabad, and Surat, India
- Researched on the current recyclable waste collection system in formal and informal sector in the Gujarat state by visiting the collection sites, and interviewing workers.
- Visited the project development site of hazardous and non-hazardous waste treatment facility, and interviewed residents in the surrounding community on the current social and economic state of the community.
- Analyzed the social, economic, and environmental impacts of introducing an integrated waste management system between formal and informal sectors, and developing the agricultural land for a new waste treatment facility.

4) **Preparatory Survey on BOP Business on Improving Maternal and Child Nutrition through Locally-Developed Food in Bangladesh**
September 2012-March 2014
Marketing Researcher, Dhaka, Bogra, and Chittagong, Bangladesh
- Conducted marketing survey in Dhaka, Bogra, and Chittagong, Bangladesh by visiting households in rural and urban areas.
- Organized test sales of pilot products developed by the client in Dhaka, Bogra, and Chittagong, and collected over 200 customer reactions on the product by conducting surveys.
- Proposed workable business model in Bangladesh to the client by negotiating with potential local partners.

5) **Preparatory Survey on BoP business on improving nutritional status of Zambia using Spirulina**
December 2013-Current
Project Manager, Lusaka, Choma, and Katete, Zambia
- Managed the project by organizing public and private stakeholders in Zambia and Japan, overseeing two Japanese and five local staff, reporting to donors, and keeping financial records.
- Conducted market and consumer research by visiting urban and rural markets, and interviewing mothers or care takers at households with different income levels in urban and rural areas.
- Developed a research partnership with School of Agriculture at University of Zambia to conduct a pilot production of spirulina and proximate analysis on the trial type of spirulina food.

6) **Preparatory Survey on BOP business on High-Value Added Skin Care Product Business by Using Surplus Agricultural Crops to Benefit Small Scale Farmers and Women**
March 2014-Current
Base Oil Ingredient and Marketing Researcher, Nairobi, Thika, and Kisumu, Kenya
- Conducted researches on potential oil ingredient extracted from agricultural products in Kenya, such as avocado, macadamia nuts, jojoba, and soya beans, to find out the production size and oil quality.
- Negotiated with potential oil provider in Kenya on business partnership for the client.
- Developed research model on measuring the socio-economic impact on local farmers by launching a skin care business using natural crops grown in Kenya.
- Managing the project as the project manager from January 2015.

Also participated in projects in Indonesia (2008), Viet Nam (2009-2010), Cambodia (2010) as a researcher, and participated in projects in Iraq (2009-2010), Egypt (2008 and 2009), and Cambodia (2008) as a project coordinator.

KAZUYA MASUDA

CONTACT INFORMATION

National Graduate Institute for Policy Studies
7-22-1 Roppongi, Minato-ku, Tokyo
106-8677, Japan

Email: phd11203@grips.ac.jp
Phone: +81 (90) 3237-4421

EDUCATION

PhD Candidate, Development Economics, National Graduate Institute for Policy Studies, September 2015

EMPLOYMENT

Research Fellow, Alliance Forum Foundation, Tokyo, November 2012-present
M.A., International Development Studies, National Graduate Institute for Policy Studies, September 2011
B.A., Economics, Keio University, included one academic year at University of California, Irvine, March 2010
Teaching Assistant, National Graduate Institute for Policy Studies, April 2012-present
Research Assistant, National Graduate Institute for Policy Studies, October 2011-present
Internship, Japan International Cooperation Agency (JICA), Lilongwe, Malawi, August-September 2011
Internship, Alliance Forum Foundation, Tokyo, July 2009-May 2010
Research Assistant, Japan External Trade Organization, Los Angeles Centre, March-July 2009

RESEARCH INTEREST

Development Economics, Economics of Education, Labor Economics, Health Economics

PUBLICATION

· Spirulina Effectiveness Study on Child Malnutrition in Zambia (2014) in "Turning Rapid Growth into Meaningful Growth: Sustaining the Commitment to Nutrition in Zambia". IDS, Brighton, UK.

RESEARCH PAPERS

· Evaluating the Impact of Universal Primary Education Policy on Completion of Primary School in Uganda, Applied Development Research, National Graduate Institute for Policy Studies, August 2011
· Effect of Girls' Education on Adolescent Pregnancy: Evidence from the Introduction of Universal Primary Education in Uganda, July 2014

HONORS AND AWARDS

GRIPS Fellowship, 2011-present

PRESENTATION

The 3rd, Keio Education Economics, and Policy Conference, Tokyo, December 2014
The 14th, East Asia Economic Association Annual Convention 2014, Bangkok, November 2014
